# Organic Bioelectronics Development in Italy: A Review

**DOI:** 10.3390/mi14020460

**Published:** 2023-02-16

**Authors:** Matteo Parmeggiani, Alberto Ballesio, Silvia Battistoni, Rocco Carcione, Matteo Cocuzza, Pasquale D’Angelo, Victor V. Erokhin, Simone Luigi Marasso, Giorgia Rinaldi, Giuseppe Tarabella, Davide Vurro, Candido Fabrizio Pirri

**Affiliations:** 1 Chilab–Materials and Microsystems Laboratory, Department of Applied Science and Technology (DISAT), Politecnico di Torino, Via Lungo Piazza d’Armi 6, 10034 Turin, Italy; 2 Institute of Materials for Electronics and Magnetism, IMEM-CNR, Parco Area delle Scienze 37/A, 43124 Parma, Italy; 3 Camlin Italy Srl, Via Budellungo 2, 43124 Parma, Italy; 4 Center for Sustainable Future Technologies, Italian Institute of Technology, Via Livorno 60, 10144 Turin, Italy

**Keywords:** Organic Bioelectronics, biosensing, neuromorphic

## Abstract

In recent years, studies concerning Organic Bioelectronics have had a constant growth due to the interest in disciplines such as medicine, biology and food safety in connecting the digital world with the biological one. Specific interests can be found in organic neuromorphic devices and organic transistor sensors, which are rapidly growing due to their low cost, high sensitivity and biocompatibility. This trend is evident in the literature produced in Italy, which is full of breakthrough papers concerning organic transistors-based sensors and organic neuromorphic devices. Therefore, this review focuses on analyzing the Italian production in this field, its trend and possible future evolutions.

## 1. Introduction

Organic Bioelectronics is the field of study that is focused on the development of organic electronic devices able to translate signals and functions between the biological word and the electronic one. First, we need to underline what we imply with the term “Organic Bioelectronics”. In the case of “Organic”, everything is clear: we want to make an overview of devices based on organic materials. Instead, “Bioelectronics” is a very general term and it must be clarified. While conventional electronics mainly rely on electron transport, biological systems commonly exchange signals via the transport of ions or molecules. The goal of this research field is therefore the integration of organic materials with biological systems for the development of novel functional and biocompatible electronic devices. In this review, we want to consider several essential features of organic electronic devices for their possible use in biosensing (with possible interfacing with traditional electronic circuits and systems), in brain-mimicking elements (artificial synapses and neurons, allowing for learning according to bio-plausible algorithms) and as interfacing elements between traditional electronic systems (computers) and the brain. In this review, we will consider mainly the works developed in Italy. However, a direct comparison with the main achievements in research groups outside Italy will be also carried out.

In recent years, organic (semi-)conductors have emerged as the materials of choice to bridge the two worlds, owing to their ability to sustain both ionic and electronic transport, to their biocompatibility and to the possibility of tailoring their properties by careful molecular design.

**Figure 1:**
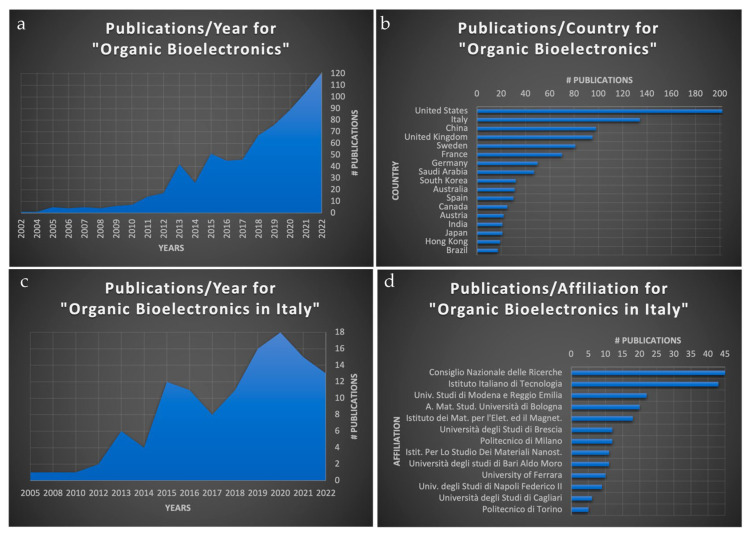
(**a**): The number of publications per year considering the keyword “Organic Bioelectronics” in the last two decades (source scopus.com, accessed on 11 January 2023); (**b**): the number of publications per country considering the keyword “Organic Bioelectronics” in the last two decades (source scopus.com); (**c**): the number of Italian publications per year considering the keyword “Organic Bioelectronics” in the last two decades (source scopus.com); (**d**): the number of Italian publications as a function of the authors’ affiliation considering the keyword “Organic Bioelectronics” in the last two decades (source scopus.com).

An analysis of the literature production in terms of publications per year, during the last two decades, shows a clear growth trend using the keyword “Organic Bioelectronics” (Figure 1a). If this trend is analyzed by focusing on the different countries, we find Italy at the second place just after the United States (Figure 1b). These data confirm the important contributions that the Italian researchers provide to Organic Bioelectronics. Moreover, considering the ratio between the number of Italian researchers with respect to other countries such as the United States, it is evident that Italy has the highest concentration of works in the field.

The extrapolated data for the Italian literature production per year (Figure 1c) show a similar trend with respect to the worldwide one, with the same picks except for the 2022, but this year can be considered underestimated due to the pending indexing of the Scopus search engine. Finally, the extrapolation of data from Figure 1c, subdivided by affiliation, shows that The National Research Council (CNR) is the first in terms of publications in the field, followed by the Italian Institute of Technology (IIT) (Figure 1d). The CNR is a generalist institution with more than 8000 employees—it includes different research groups afferent to different institutes that cover topics from basic science to application and industrial-oriented development. The CNR has a strong tradition in the field of bioelectronics and has been involved in several initiatives aimed at advancing the development of innovative devices and techniques for healthcare applications. For example, researchers at CNR have developed advanced biosensors for monitoring and diagnosing various conditions, such as cancer and neurodegenerative diseases [1], and have worked on developing novel materials and techniques for neuromorphic devices [2].

The CNR’s contribution from Istituto dei Materiali per l’Elettronica ed il Magnetismo (IMEM-CNR) accounts for almost half of the total CNR’s production, focusing on the materials and the physics of bioelectronic devices [3] as well as the testing of the devices for in vivo or agriculture applications [4,5]. The IIT is a more industrial development-oriented institution with the aim to contribute to the economic development of the country. To accomplish this mission, the works from IIT researchers have a high technology readiness level (TRL), typically from 4 to 6. In this view, IIT is more oriented to the application, but on the other hand, new and interesting works are arising at a lower TRL from the biological field, i.e., interaction with cells to optimize the interface for electrical stimulation [6].

Different and significant works came also from Italian academics, particularly in the development of innovative devices; here are a few notable examples: at the University of Brescia and University of Bari, researchers have been involved in the development of innovative devices on biodegradable and compostable substrates [7] or in the use of green materials and technologies for bioelectronics [8]. At the University of Modena and Reggio Emilia and University of Ferrara, researchers have focused on developing new materials and techniques for neuromorphic devices for label-free dopamine detection [9] and for in vivo electrophysiological signal studies [10]. Researchers from the University of Cagliari are instead developing new biosensors and devices for cellular analysis [11]. At the Polytechnics of Milano and Torino, researchers have been involved in the development of advanced technologies for new chip design and functionality [12] and for integration and sensing in microfluidic systems [13].

It is worth mentioning another important Italian research center, the Bruno Kessler Foundation (FBK). While FBK is mainly specialized in the development of silicon-based devices, they recently started working on Organic Bioelectronics as well, and contributed to the field with a noteworthy review on organic electrochemical transistors for in vivo applications [14].

The described list depicts a literature production that is widespread over the whole country, from north to south. In honor of the truth, a maximum concentration was reached in the Central Italian Region, i.e., Emilia Romagna in the cities of Bologna, Parma and Modena.

In this review, the complete Italian scenario on Organic Bioelectronics is presented, considering the last and most significant works in the field collected and subdivided them into two main categories: (i) transistor-based sensors; (ii) neuromorphic devices.

Section 2 presents the most exploited materials for the fabrication of organic electronic and bioelectronic devices, comprising conductive polymers as well as organic small molecules.

In Section 3, the latest development concerning organic transistors for sensing applications are presented. The first part of this section will focus on device engineering (i.e., fabrication techniques, modelling), while the following subsections will be dedicated to the different applications. Firstly, immuno-affinity-based and nucleic-acid hybridization-based biosensors are going to be presented, followed by the application of organic transistors for ions and pH sensing and the development of flexible and wearable devices. The last part of this section will focus on the application of organic transistors for in vivo and cell monitoring.

Section 4 will focus on devices for neuromorphic applications. The term “neuromorphic” implies the possibility of reproducing several features of the nervous system and brain. In the first part of this section, organic memristive devices and organic transistors applied as neuromorphic devices are going to be presented. We will consider such features as supervised learning (artificial neuron networks), unsupervised learning (spike-timing-dependent plasticity (STDP), frequency-driven plasticity, short-/long-term potentiation/depression), and model systems allowing for classic conditioning (Pavlov dog). The section will conclude with a description of the experimentally realized system, allowing for the coupling of neuromorphic memristive devices with live neurons, directly demonstrating that these devices can be considered as artificial synapse analogs and providing a significant step towards the realization of nervous prostheses.

## 2. Materials

Since their serendipitous discovery by Shirakawa et al. [15], conductive polymers (CPs) are disclosed as materials able to combine the mechanical features of plastics with the electrical conductivity properties typical of metallic species [16]. Specifically, the electronic currents’ conduction is allowed by the delocalized π-electrons along the polymeric backbone, due to the alternance of single and double bonds between the carbon atoms [16,17,18]. In addition, the CPs’ chains can reversibly transform between oxidation states by chemical or electrochemical processes. These redox pathways are accomplished by the involvement of mobile dopant ions, of which counterbalance the generation of charge carriers such as polarons, bipolarons, solitons, etc., induced by the arrangement of the polymer chain in the different redox configurations [19,20]. At higher doping levels, these charged molecular defects introduce energy states within the electronic band gap, tuning the charge transport properties of the material into a quasi-metallic regimen [19]. Moreover, the ion exchange mechanisms induced by the involvement of the dopant ions during the redox processes allows for CPs to have ionic conduction properties [21].

These overall peculiarities make CPs particularly suitable to be used in organic electronic devices as active materials [22,23] or in combination with other elements [24,25].

For their exploitation, CPs are commercially available or can be synthesized by chemical, electrochemical, emulsion, interfacial, photochemical and solid-state polymerization methods [18]. The simplest method is the chemical oxidative polymerization that is accomplished by oxidizing monomers or polymer precursors with an oxidizing agent, such as ammonium persulfate (APS) or ferric chloride (FeCl_3_). On the other hand, the electrochemical polymerization routes allow for the production of electrically and electrochemically active CPs films with a low amount of monomer and short duration of the polymerization process.

All these synthetic routes inherently require anionic dopants to neutralize the positively charged CP backbone [26]. The choice of preparation method and dopant agents is crucial to modulate a series of factors such as water or organic solvents’ solubility, processability, mechanical, electrical and biological properties of the resulting CP-based systems [27,28,29,30,31], tailoring the specific applications of the produced materials.

Among CPs, poly (3,4-ethylenedioxythiophene) (PEDOT) and polyaniline (PANI) are demonstrated to be the main candidates as active materials for electronic devices, owing to peculiar features such as designability, charge transport properties, mechanical flexibility, electrochemical activity, high signal transduction, dimensional durability and processability [27,32,33,34].

PEDOT molecules can encompass two oxidation processes, such as the conversion from the neutral state to polarons and from polarons to bipolarons [35].

The charge transport properties of PEDOT are favored by the linear, rigid molecular conformation [36]. However, PEDOT by itself is typically insoluble in water or common organic solvents [37]. Therefore, to improve its processability and solubility in water, the synthesis of PEDOT is accomplished in the presence of poly(4-styrenesulfonate) (PSS) to produce a stable dark-blue color aqueous dispersion containing both polymers (PEDOT:PSS). Commercially available PEDOT:PSS dispersions are acknowledged to possess remarkable conductivity, high work function, excellent chemical and electrochemical stability, optical transparency, biocompatibility, and so on [17]. Moreover, the compatibility with deposition techniques such as drop-casting, spin-coating and spray-coating make PEDOT:PSS dispersions particularly attractive for the fabrication of OEDs [17,37].

In the CPs scenario, PANI is a well-known conductive polymer that has a large use in multiple electronic applications, from material for pH sensors to conductive element for bio-scaffolds. It exists in 3 oxidation states, such as reduced leucosmeraldine (L), semi-oxidized emeraldine (E) and oxidized pernigraniline (P). The passage between these various configurations could be accessed by inducing reversible redox reactions. Independently of the oxidation state, the base forms of PANI are electrically insulating. However, the conversion of emeraldine base (EB) to emeraldine salt (ES) form of polyaniline can increase the electrical conductivity of PANI from insulator (σ < 10^−10^ ohm^−1^cm^−1^) to metallic (σ ≈ 10^+1^ ohm^−1^cm^−1^), simply through protonation processes. Therefore, the protonated semi-oxidized state ES form of PANI is constituted by a delocalized poly(semiquinone radical cation) with a polaron conduction band, of which allows for an efficient electron transfer. As discussed above, the incorporation of counterions (anions or cations) within the PANI matrix during the redox processes can guarantee to such a material both electric and ionic conductivity in response to electrical stimuli. These mechanisms are similar to the reactions that take place in organs involving some neural functions, making PANI systems valid candidates in memristive technologies.

Among 𝜋-conjugated organic polymers, poly(3-hexylthiophene) (P3HT) is another material extensively studied for applications in Organic Field-Effect Transistors (OFET) and solar cells. The rigid backbone of this polymer allows for the formation of planar chain segments as well as aggregates and large crystallites along which fast charge transport is obtained. Depending on processing parameters such as solvent, deposition techniques and thermal treatments, and on material parameters such as molecular weight and regioregularity, it is possible to tune the density and dimensions of these crystallites [38,39]. A fundamental study by Sirringhaus et al. [40] demonstrated how the organization of P3HT in lamellar structures normal to the substrate increases mobility by more than two orders of magnitude compared to lamellar stacking parallel to the substrate. The increased mobility was attributed to the two-dimensional nature of polaronic charge carriers formed in the inter-chains 𝜋-𝜋 stacking plane.

Apart from CP, a lot of attention has been focused also on the exploitation of organic small molecules as active materials in organic electronic devices. Small molecules are able to form tightly packed molecular crystals kept together by Van der Waals interaction. Similarly to inorganic crystals, charge transport in these molecular crystals takes place by the delocalization of charge carriers in a band of states. However, the weak interactions holding together molecular crystals result in an important contribution of lattice vibrations (phonons) which scatter the charge carriers thus limiting the overall mobility (typically in the order of $1\;{\mathrm{cm}}^{2}V^{-1}s^{-1}$ at room temperature) [41]. Pentacene deposited by high-vacuum evaporation from a Knudsen cell has been the reference molecule for the realization of organic devices for a long time [42,43]. Recently, 6,13-Bis(triisopropylsilylethynyl)pentacene (TIPS-pentacene) has attracted more and more attention. This is a derivate of pentacene, functionalized with side chains specifically designed to allow for dispersion in organic solvent and to improve the 𝜋-𝜋 stacking of molecules in thin films. The possibility to solution-process this material, combined with its high mobility, resulted in the realization of several high-performing devices [44,45]. A more detailed review of the role of small molecule semiconductors for OFET-based biosensors may be found in the work of Tao et al. [42].

## 3. Transistor-Based Sensors

### 3.1. Device Structure and Working Principles

**Figure 2:**
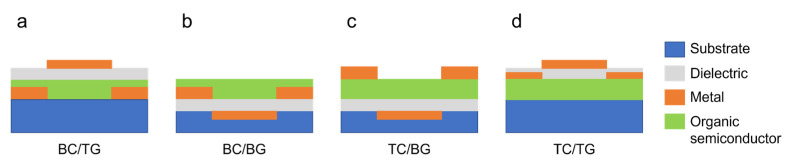
Different OFET architectures. (**a**): Bottom Contact–Top Gate; (**b**): Bottom Contact–Bottom Gate; (**c**): Top Contact–Bottom Gate; (**d**): Top Contact–Top Gate.

In OFETs, similarly to inorganic FETs, the semiconductor layer is separated from the gate electrode by an insulating dielectric layer. In planar devices, different configurations are available depending on the position of source, drain and gate electrodes relative to the organic semiconductor layer, as shown in Figure 2. Transport in organic semiconductors (OSC) is mainly based on the hopping of charge carriers between sites with energy distributed within a Gaussian density of states (DOS). In organic FETs, charge carriers are injected in the OSC from the electrodes, and the current can be modulated by tuning the charge density at the semiconductor/insulator interface varying the gate voltage 𝑉𝑔𝑠. The gradual channel approximation can be exploited to model the drain current of the devices equivalently to the case of inorganic semiconductors [41]:

(1) $$I_{ds}=\mu C_{i}\frac{W}{L}\left(\left(V_{gs}-V_{th}\right)V_{ds}-\frac{1}{2}V_{ds}^{2}\right)$$

where 𝐶_𝑖_ is the capacitance per unit area of the gate dielectric, and 𝑉_𝑡ℎ_ the threshold voltage of the device. When |𝑉_𝑑𝑠_| ≪ |𝑉_𝑔𝑠_| − |𝑉_𝑡ℎ_| and |𝑉_𝑔𝑠_| > |𝑉*_th_*|, Equation (1) reduces to:

(2) $$I_{ds}=\mu_{lin}C_{i}\frac{W}{L}\left(V_{gs}-V_{th}\right)V_{ds}$$

which describes the drain current in a linear regime. If |𝑉_𝑑𝑠_| > |𝑉_𝑔𝑠_| − |𝑉_𝑡ℎ_|, on the other hand, a depletion region arises at the interface between the organic semiconductor and the drain electrode, and the channel is in pinch-off. In this situation, the maximum voltage drop along the channel is dictated by |𝑉_𝑔𝑠_|−|𝑉_𝑡ℎ_|, the current becomes independent on the drain voltage and the saturation regime is reached:

(3) $$I_{ds}=\frac{1}{2}\mu_{sat}C_{i}\frac{W}{L}{\left(V_{gs}-V_{th}\right)}^{2}\;$$

**Figure 3:**
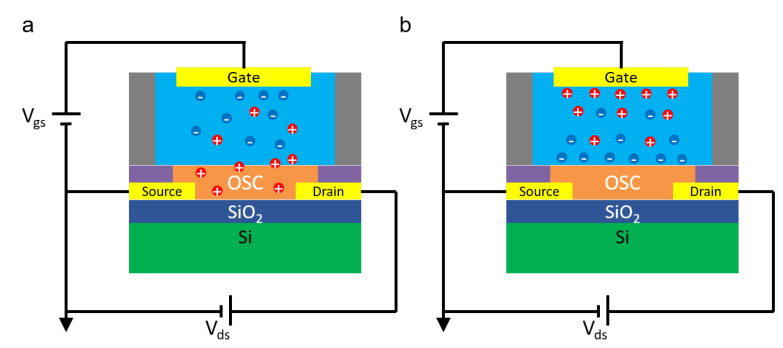
(**a**): Schematic architecture of an OECT. Application of a positive gate voltage induces cation penetration in the OSC with consequent electrochemical doping. (**b**): Schematic architecture of an EGOFET. Application of negative gate voltage induces the accumulation of anions (cations) in the EDL at the semiconductor/electrolyte interface, which in turn induces an accumulation of holes in the semiconductor.

This set of equations, even though originally obtained for solid-state devices, is suitable to describe the operation of electrolyte-gated transistors as well. Similarly to Ion-Sensitive Field-Effect Transistors (ISFETs) [46,47], these devices are interfaced with a liquid electrolyte at the gate. The main difference between electrolyte-gated transistors and ISFETs resides in the lack of a gate dielectric, which is completely replaced by the electrolyte. The gate electrode is then immersed in the liquid (Figure 3). When using an organic semiconductor in direct contact with an electrolyte, two different operation modes are possible. If the OSC is permeable to ions and capable to withstand mixed electronic/ionic conduction, an electrochemical operation is obtained, and the material is classified as an organic mixed electronic-ionic conductor (OMIEC). In this case, the electrochemical doping/de-doping of the semiconductor controls the charge carrier density in the device channel, and the device is an Organic Electrochemical Transistor (OECT) (Figure 3a). When the OSC is not permeable to ions, instead an electrical double layer (EDL) is formed at the semiconductor/electrolyte interface. The voltage drop at the solid/liquid interface allows to control the charge carrier density, and an Electrolyte-Gated Organic Field-Effect Transistor (EGOFET) is obtained (Figure 3b). This classification, however, is not so clear-cut in reality. For example, ionic penetration in the channel of EGOFETs may take place under certain conditions, and a degree of current modulation due to field effects may be expected in OECTs [48,49].

As already mentioned, OECTs rely on the injection of ions from the gate electrolyte in the semiconductor to modulate the charge carriers’ density in the channel. The electrochemical doping/de-doping mechanism that takes place throughout the volume of the channel allows for the ability to reach higher transconductances (due to the volumetric capacitance contribution) compared to field-effect devices, and to operate at a low bias voltage (typically |𝑉_𝑔𝑠_| ≤ 1 V). The ability to work with low applied potential and to efficiently transduce ionic signals to electronic ones make these devices particularly promising for biosensing applications and bio-interfaces.

On the other hand, in EGOFETs, the control over the charge carrier density in the channel is achieved via field effects due to the formation of an electrical double layer at the semiconductor/electrolyte interface. The structure of this EDL is usually described in terms of the Gouy–Chapman–Stern model [50]. When a negative (positive) 𝑉_𝑔𝑠_ is applied at the gate electrode, anions (cations) are pushed towards the device channel. The accumulation of anions (cations) at the solid/liquid interface gives rise to a negative (positive) surface charge density on the liquid side which, in a p-type (n-type) semiconductor, is compensated by the accumulation of holes (electrons) in a thin layer of material close to the surface. The EDL capacitance 𝐶_𝑒𝑑𝑙_ (usually on the order of tens of $\mu F/{\mathrm{cm}}^{2}$ [50]) therefore governs the response of the device, and it replaces the dielectric capacitance in Equations (1)–(3). The higher value of 𝐶_𝑒𝑑𝑙_ compared to the one achievable with solid dielectrics (on the order of tens of nF/cm^2^) allows for low operating voltages, with 𝑉_𝑔𝑠_ typically below 1 V for application in aqueous solutions. Moreover, the field-effect operation mode allows for EGOFET to reach a faster response time, but it limits their transconductance compared to OECTs, where the contribution of a volumetric capacitance plays a pivotal role [48].

The mode of operation of the devices can be controlled by varying the polymer side chains [48,51]: polar side groups in fact render the polymer surface hydrophilic, facilitating ion diffusion in the bulk of the semiconductor and thus allowing for electrochemical operation. On the other hand, non-polar side chains prevent the diffusion of ions inside the polymer matrix, ensuring a dominating field-effect operation mode. However, electrochemical doping may take place also in hydrophobic polymers depending on the operating conditions [51].

A role of paramount importance in the operation of both OECTs and EGOFETs is played by the gate electrode. When using a noble metal polarizable electrode, an EDL arises at the interface between the metal and the electrolyte [50]. This results in capacitance 𝐶_𝑔_ in the series with one of the semiconductor channels (𝐶_𝑐_). In order to keep reliable control over charge carrier density in the channel, the contribution of the gate electrode capacitance must be minimized by granting that 𝐶_𝑔_ ≥ 10 × 𝐶_𝑐_ [52]. Using a non-polarizable gate electrode like Ag/AgCl, on the other hand, grants that most of the voltage drop arises at the semiconductor interface [52].

The prevailing success of organic transistors in biosensing/bio-interfacing applications stems from the improvement of performances and reliability achieved in recent years. The application of different materials, geometries and fabrication techniques, together with a deeper understanding of device physics, allows for the tailoring of the device performances to the specific applications, reaching unprecedented results. The Italian production in this field reflects a generalized trend, with the exploration of materials ranging from small molecules and polymeric OSC [39,53,54,55,56], to polymeric OMIECs [57,58,59]. The use of OSCs or OMIECs gated via an electrolyte has proven to be a successful configuration for applications in bioelectronics [60]. As previously discussed, the resulting EGOTs are usually classified in terms of their operational regime.

Focusing on bio/chemical sensing applications, the main drawback of EGOT configuration resides in the inherent operational instability of the OSC/OMIECs operating in direct contact with an electrolyte (typically an aqueous solution) [61]. A possible solution to this problem is the exploitation of a different configuration, namely the extended gate (or floating gate) configuration [62]. In this configuration, a typical OFET structure is exploited. The gate electrode acting as a sensing element (floating gate) is extended far away from the OSC channel and put in contact with the electrolyte, thus avoiding the direct contact between the OSC and electrolyte [62]. A similar configuration is represented by the Organic Charge-Modulated Field-Effect Transistor (OCMFET) [54].

From a technological point of view, fabrication techniques derived from silicon technology are exploited [39,57,63,64,65,66], as well as 2D and 3D printing technologies [7,54,67,68,69,70,71]. Moreover, increasingly accurate models have been developed in order to better understand the physical mechanisms governing the device operations, both in terms of the electrolyte-gating working mechanisms and in terms of the biosensor’s response to a given analyte [55,56,59,72]. In the following subsections, the cutting-edge progress achieved by the Italian Organic Bioelectronics community concerning the fabrication and modelling of novel organic transistor-based sensors will be described.

Finally, regarding device applications, the sensing of bioanalytes by electrolyte-gated organic transistors (EGOTs) basically exploits electro-chemical approaches. EGOFET OECT architectures are used to transduce chemo-physical phenomena (i.e., redox reactions or the generation of electrical double layers) taking place at the decorated gate electrode surface in the presence of bioanalytes. Such biosensors base their operation on recognition mechanisms involving a given bioanalyte and, depending on its chemical nature, enzymes that are able to catalyze electrochemical reactions or highly affine recognition systems for target molecules, such as antibodies, aptamers and nanobodies. The latter are specific systems capable of implementing a selective binding with related biomolecules. In enzymatic sensing, oxidases are used to decorate device interfaces in order to catalyze the conversion of a bioanalyte upon the reduction in selected enzymes that, in turn, reactivate from the reduced state by generating an amount of hydrogen peroxide proportional to the concentration of the reacting bioanalyte. The electrochemical oxidation of H_2_O_2_ at a platinum electrode is then transduced by the organic-based transistor channel. Enzymatic approaches are well suitable for the real-time monitoring of bioanalytes. Indeed, the detection of bioanalytes by recognition systems exploits their interaction with the related ligands decorating a device interface (generally, the gate electrode). Accordingly, the recognition is mostly implemented by evaluating the binding events’ efficiency upon performing some comparative measurements prior and after immobilization of the bioanalyte on the decorated interface.

Since the beginning of the previous decade, several groups operating in the field of organic electronics have contributed to the sensing of bioanalytes performed via transistor architectures, mainly exploiting transducers that are made of organic semiconductors [73,74,75], even through organic channel functionalization [76], but also oxides [77] and inorganic conductors [78] or devices with a real 3D structure monolithically fabricated by additive manufacturing approaches [71]. Pioneering works exploit organic transistors with unfunctionalized device interfaces to measure, for instance, variations in pH using a dual gate field-effect device [79]; to implement enantioselectivity by a chiral bilayer field-effect device for the detection of a monoterpenoid, i.e., the citronellol (beta-isomer) [80]; to selectively detect a neuro-transmitter, the dopamine, in the presence of ascorbic and uric acids as interfering molecules and an all-PEDOT:PSS-based OECT [81]; and to detect, in real-time, drug delivery systems (specifically, micro vesicles made of liposomes) by an OECT combined with microfluidics to implement the lab-on-a-chip approach, thus showing the extent at which organic devices are suitable for integration strategies [82].

### 3.2. Transistor Fabrication and Modelling

#### 3.2.1. Device Micro-Fabrication and Downscaling

A standard approach for the fabrication of EGOTs relies on the application of microfabrication processes derived from silicon technology. The bottom contact configuration is the most commonly exploited, and different substrates such as Si/SiO_2_ wafer, glass, Kapton^®^ or other polymeric materials may be used [39,57,65,66]. The first fabrication step consists of the definition of source and drain (S/D) electrodes. Au is typically chosen as an electrode material due to its high work function, which allows for an easy injection of holes in the Highest Occupied Molecular Orbital (HOMO level) of the p-type OSCs/OMIECs generally adopted. The S/D fabrication starts with a thin film deposition (Cr or Ti are used as an adhesion layer prior the Au deposition), followed by a photolithographic step and wet etching [39]. Alternatively, a lift-off process may be employed, starting with photolithography followed by thin film deposition and lift-off of the photoresist [59].

**Figure 4:**
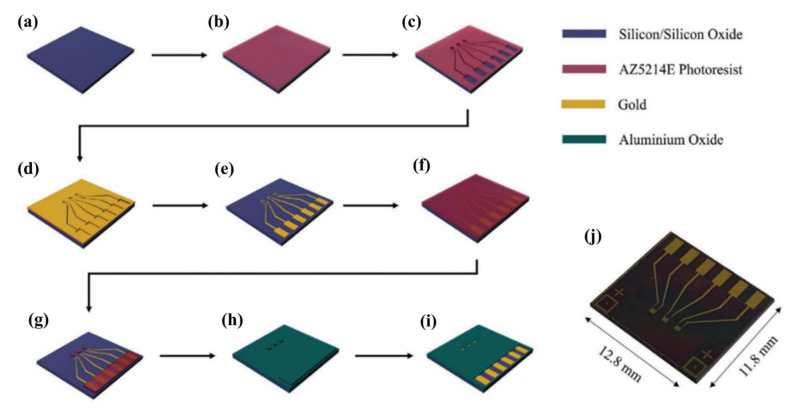
Schematic example of a standard micro-fabrication process employed for EGOT fabrication. (**a**–**e**): lift-off process for the definition of source and drain electrodes; (**f**–**i**): lift-off process for the definition of contacts passivation. (**j**) Optical picture of the final device. Copyright (2022) Wiley. Used with permission from M. Segantini et al., “Investigation and Modeling of the Electrical Bias Stress in Electrolyte-Gated Organic Transistors,” Adv. Electron. Mater., vol. 8, no. 7, 2022, Wiley [59].

The second step in the fabrication is the deposition of a passivation layer. This is fundamental to prevent short circuits during the operation of the transistor in contact with an electrolyte. A passivation layer may be deposited via chemical vapor deposition (CVD) [83], e-beam evaporation [59] or spin-coating [66], depending on the material of choice. A lithography/etching process (or alternatively a lift-off process) is necessary in this step in order to open windows in the passivation. These are needed to allow for the OSC/OMIEC to contact the electrodes, and to connect the contact pads with the external world. The described process is summarized in Figure 4, where a double lift-off process was employed to define S/D electrodes and passivation windows (in this case, Al_2_O_3_ deposited via e-beam evaporation) [59].

The final step in the device fabrication consists of the deposition of the organic (semi-) conductor. Spin-coating is the most common technique, allowing for good control over film thickness without strong limitations in the choice of the solvent [39]. Again, the active material may be patterned by means of an etching or lift-off process [57,65].

While the process described above is well suited for the fabrication of devices with channel lengths ranging from a few micrometers to hundreds of microns or longer, more and more interest is being focused on the downscaling of transistor dimensions. From a bioelectronics point of view, different reasons are driving this interest. Shorter channel devices will allow for a higher integration density and operating speed, both being necessary for the fabrication of compact high-performance bioelectronics chips. Moreover, bio-interfacing and the recording of biological signals may greatly benefit from sensors able to collect signals with precise spatio-temporal resolution. In this contest, three different approaches for the downscaling of OECTs have been explored with the participation of Italian researchers.

D’Angelo et al. demonstrated a sub-micrometric channel OECT by employing the electromigration-induced break junction (EIBJ), a technique derived from molecular electronics to fabricate S/D electrodes with the separation of a few nanometers without expensive e-beam lithography processes [63]. The fabricated nanogap OECT demonstrated superior performances in the amplification of fast, varying signals. Indeed, the reduced volume of the transistor channel allowed for minimizing the role of ion diffusion, granting a superior speed of device operation.

A different approach was exploited by Koutsouras et al. who developed a vertical channel OECT (vOECT) [64]. An electrodeposited PEDOT:PSS layer sandwiched between the source and drain electrodes acted as an active device channel, with the length defined by the film thickness. By varying the lateral geometrical dimensions of the device, the authors demonstrated ultra-high transconductance in the range of hundreds of mS for the larger geometries. A similar approach has also been exploited by both Italian and non-Italian research groups for the fabrication of vOECT-based n-type organic transistors and complementary circuits [84,85]. Moreover, researchers from FBK recently reviewed the impact of planar and vertical geometries on OFET performances for flexible electronics [86]. In particular, they highlighted the need for more studies to focus on vOFETs, which can provide a feasible solution for developing thinner and more compact devices.

Finally, Mariani et al. realized an all-PEDOT:PSS OECT with needle-type architecture [87]. They exploited single- and double-barrel carbon nanoelectrodes to fabricate a nanometer-sized OECT and gate for sensing with a high spatial resolution of dopamine. The device showed cell-compatible dimensions and compatibility for future in vivo studies.

#### 3.2.2. Device Printing

Printing techniques are emerging as a straightforward choice for the fabrication of organic electronics devices. Indeed, the use of solution-processable organic semiconductors in combination with printing processes allows for cost-effective fabrication over a large area and on flexible substrates. The combination of these characteristics with the biocompatibility provided by organic semiconductors and polymeric materials opens a plethora of possibilities for applications in bioelectronics [60].

A comparison between inkjet printing and spin-coating of organic semiconductors has been performed by Blasi et al. for the fabrication of P3HT-based EGOFETs [67]. P3HT was deposited via inkjet printing on a photolithographically patterned source and drain gold electrodes. The printed devices showed better long-term stability in terms of maximum drain current and threshold voltage compared to spin-coated ones. These performances were ascribed to a lower energetic disorder in the (although less numerous) crystalline domains of the printed films. The more-ordered crystallites were attributed to the use of a higher boiling point solvent for inkjet printing with respect to spin-coating (ortho-dichlorobenzene vs. chlorobenzene), rather than to the deposition technique itself.

In 2018, Lai et al. at the University of Cagliari fabricated a floating gate Organic Charge-Modulated Field-Effect Transistor (OCMFET) by the means of large area processes over flexible substrates [54]. In particular, they exploited the inkjet printing technique in combination with chemical vapor deposition (CVD)—both processes being easily scalable to the industrial level. The OCMFET was characterized by a floating gate configuration, with the gate electrode, source and drain electrodes and organic semiconductor patterned via the inkjet printing of different materials (silver ink for the gate, PEDOT:PSS for source and drain and tips-pentacene as the organic semiconductor), and the polymeric gate dielectric (parylene-C) deposited via CVD. By opportunely connecting the floating gate of the transistor to a temperature-dependent or to a pressure-dependent capacitive element, the authors demonstrated the viability of the device as a temperature or pressure sensor in the range of interest for bioelectronic applications. In a subsequent study, they further developed the OCMFET by coupling the device with a graphene floating gate [68]. In this work, functionalization of the graphene electrode with sensing peptides bearing a pyrene moiety was exploited in order to realize a pH sensor.

Bertana et al. took advantage of rapid prototyping techniques to fabricate a fully 3D-printed OECT [71]. The device fabrication was carried out by the means of 3D Stereolithography (3D-SL), exploiting a composite photocurable resin as OMIEC for the active transistor channel. The resin was obtained from two components: poly(ethylene glycol)diacrylate (PEGDA) as photocurable material and PEDOT:PSS as an ionic–electronic conductive material. The treatments necessary for the resin preparation resulted in the partial removal of PSS which was replaced by a PEDGA matrix surrounding the PEDOT crystallites. After a thorough characterization of the electrochemical and physical properties of the material, the device was applied as a proof of concept for dopamine biosensing, displaying an enhanced sensitivity of 0.41 V/dec. The 3D printing technology employed enabled freeform fabrication of the devices, allowing for their integration in IoT and smart objects.

3D printing was also exploited by Sarcina et al. for the fabrication of a gate electrode to be exploited in combination with an EGOFET for the detection of pancreatic mucinous cyst markers, achieving a limit of detection in the zM range [88].

Screen-printing technology was instead exploited by Sensi et al. for the fabrication of flexible PEDOT:PSS-based OECTs [69]. The whole fabrication process was carried out by screen printing the different components of the device (source and drain electrodes, gate electrode, OMIEC channel) on a PET substrate. The screen-printed carbon-gate electrode was covered with an electrodeposited Au film and functionalized by the means of potential-pulse-assisted self-assembled monolayers (SAM) to investigate the device’s faradaic operation. In a later work from the same research group, the carbon gate was covered with electrodeposited Pt and functionalized with a hydrogel double layer [70]. These layers, behaving as a charge selective barrier, allowed for the specific detection of H_2_O_2_ generated as part of the uricase regeneration process during uric acid monitoring experiments.

**Figure 5:**
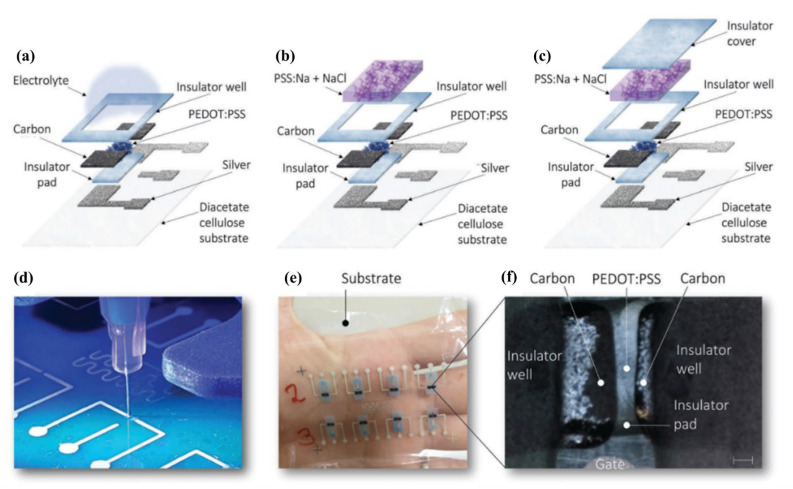
(**a**–**c**): Schematic fabrication process of fully printed, maskless OECT. (**d**): Picture of the device during the printing process. (**e**): Final devices on flexible, transparent substrate. (**f**): Micrograph of a finite device. Copyright (2022) Wiley. Used with permission from R. Granelli et al., “High-Performance Bioelectronic Circuits Integrated on Biodegradable and Compostable Substrates with Fully Printed Mask-Less Organic Electrochemical Transistors,” Small, vol. 18, no. 26, 2022, Wiley [7].

More recently, in 2022, Granelli et al. realized a fully printed OECT-based bioelectronic circuit on diacetate cellulose thin film, a compostable substrate [7]. The fabrication combined dispensing and direct writing methods for the deposition and patterning of the different parts of the transistors, avoiding material waste and resulting in a complete environmentally friendly process. The obtained transistors (Figure 5) displayed state-of-the-art performances both when integrated in a unipolar inverter and when exploited for ion detection.

While all the studies discussed so far deal with the realization of p-type transistors, Viola et al. from the Italian Institute of Technology recently demonstrated an n-type EGOT based on a printed polymer [89], which allows for the fabrication of water-gated digital electronic circuits.

#### 3.2.3. Device Modelling

Physical modelling has emerged from several points of view as a fundamental aspect in the evolution of integrated bioelectronic devices. The design of microfluidic cartridges aimed at avoiding device contaminations [13,90], the description of physical mechanisms involved in EGOT operations [59,72], the proper co-design of an electronic device and functionalization strategy [55] and the correct interpretation of a biosensor’s response at different analytes [53,56]; these are all fundamental parts of the device engineering benefiting from the development of more accurate models. In the following section, we will focus on the last three aspects, skipping the design of microfluidic devices.

The basic model describing the OECT operation was developed in 2007 by Bernards and Malliaras, and is referred to as Bernard’s model [91]. Recently, more and more accurate models have been developed [92,93]. In the Italian community, Gentile et al. provided a mathematical model describing the OECT operation for plant monitoring, where the wet fraction of the channel itself and the ion concentration may change during the operation of the device [72].

Segantini et al. focused instead their attention on the stability of EGOT (both EGOFET and OECT) when subjected to electrical bias stress (EBS) [59]. The authors exploited two closely related polythiophene (P3HT and P3CPT) for the fabrication of the different architectures, and investigated the bias-stress-induced-performance degradation in the two devices by the means of electrical, electrochemical and spectroscopic analyses. The authors then developed a mathematical model able to reproduce the device behavior, taking into account the degradation of the OSC/OMIEC subjected to EBS.

From the point of view of co-engineering EGOFET geometry and bio-functionalization, a pivotal work has been provided by Picca et al. [55]. Starting from the standard equations describing the MOSFET operation, the authors took into account the different contributions given by a bio-layer that are attached on the gate electrode or on the OSC channel. The developed model accurately describes how variations in capacitance and/or surface charges upon specific binding on the different surfaces influence, in different ways, the transfer characteristics of the transistors. In particular, while modifications of surface charge distribution are expected to be mirrored in threshold voltage shifts, a variation of capacitance in the immobilized bio-layer can give a modification of EGOFET transconductance. The research group also developed a model to prove the feasibility of detection at low concentrations in a very short time [94].

Concerning the transduction mechanism from a bio-recognition signal to an electrical one, Berto et al. provided a useful model to clarify the varying sensitivity of EGOFET at different TNFα concentrations [53]. The developed model describes the super-exponential increase in sensitivity at a low concentration as deriving from the shape of the HOMO DOS of the OSC. The EGOFET response over the whole range of analyte concentrations is then described with a combined model interpolating between a Hill-type isotherm at low concentrations and a Langmuir-type one at high concentrations. A step further in the modelling of EGOT dose curves was then performed by Urbina et al. [56]. In their work on interleukin-6 (IL-6) biosensing, the researchers demonstrated the viability of Frumkin isotherm to describe the curves obtained by both EGOFETs and OECTs. The Frumkin isotherm allowed for the clarifying of the competing or synergic phenomena affecting biorecognition at the gate electrode in terms of coverage-dependent interactions, thus providing association constant values that are more reliable than Hill’s model.

### 3.3. Catalytic and Immuno-Affinity-Based Sensors

**Figure 6:**
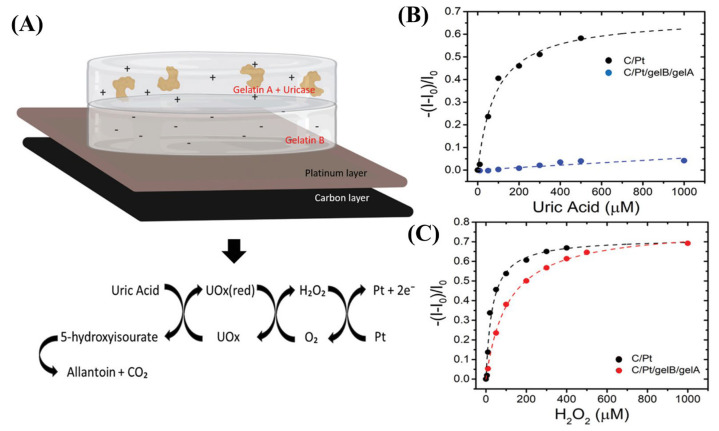
Functionalization strategy. (**A**): Top: Functionalized gate presenting a platinum layer on top of the carbon-printed gate enzyme (the cartoon representation is not to scale). Bottom: the reactions involved in the enzymatic detection of UA. (**B**): Normalized current variation at fixed VGS = 0.5 V, as a function of [UA] for OECT gated by a bare platinum gate (C/Pt, black dots) and at a platinum, further-functionalized gate with gelatin A and B (C/Pt/GelB/GelA, blue dots). （**C**): Normalized current variation as a function of [H_2_O_2_] for OECT gated by C/Pt (black dots) and by C/Pt/GelB/GelA (red dots) electrodes. Reprinted with permissions from [70].

Unfunctionalized device interfaces suffer from a lack of selectivity towards the desired bioanalytes in the presence of interfering molecules. Selectivity is a fundamental requirement for promoting reliable biosensors, and both catalytic biosensing and immunosensing are the most popular approaches in biosensing; exploiting the H_2_O_2_ production and the antigen–antibody interaction is a key factor for promoting specificity. In line with the worldwide trend regarding research activities in the field of biosensing, several analytes have been detected by Italian groups making use of transistor-like biosensors, mostly paying attention to biomolecules of interest for medical diagnostics. Catalytic biosensing has been shown to be effective for the detection of various biomarkers. For instance, biosensing based on screen-printed, flexible OECT standard architectures has been implemented by Galliani et al. for the detection of uric acid in wound exudate using bilayers of natural hydrogels (gelatin A and gelatin B) for the immobilization of urate oxidase onto platinum gate electrodes [70] (Figure 6). A limit of detection (LOD) of approximately 10 µM has been found in this case. Indeed, innovative [95] and wearable textile [96] OECT platforms have been employed to detect biomolecules. In the first case, a planar configuration is used. It consists of a PEDOT:PSS channel and an Au gate electrode covered by electrodeposited Ni/Al-Layered Double Hydroxide acting both as a matrix for functionalization by glucose and lactate oxidase and as an active layer for promoting the electrochemical oxidation of H_2_O_2_. The LOD calculated for glucose and lactate from calibration curves (linearity of device response as a function of the analyte concentration) are 2 × 10^−2^ mM and 4 × 10^−2^ mM, respectively. Indeed, the latter application regards the detection of a standard amino acid, tyrosine. The device makes use of a copper-containing oxidase, i.e., laccase, providing an LOD of 10 nM.

Some early studies on immunoaffinity sensors were aimed at demonstrating the attitude by antigen-antibody recognition at implementing a proper detection of target bioanalytes, rather than at consolidating their suitability at working in the presence of complex matrices such as body fluids. For instance, Casalini et al. have exploited single-molecule force spectroscopy to shed light on the features of binding events in the presence of two functionalization protocols involving an Au gate electrode and antibodies for interleukin-4 (IL-4, an inflammatory cytokine) [97]. They showed that the best performance in the detection of IL-4 by a pentacene-based EGOFET is realized in the correspondence of binding events that are successfully accomplished on 30% of the gate surface area. More recently, Manoli et al. coupled a multi-layer protein system with an EGOFET to show how overcoming the device’s insensitiveness to detect the desired species dissolved in electrolytic media due to electrostatic screening effects (EDL formation) limiting the operation when gate electrodes were placed at a distance higher than the Debye length (typically, ranging around 2 nm) from the organic channel [98]. The proposed approach has been shown to be effective for the detection of proteins (Avidin and Streptavidin) in PBS for a gate-to-channel distance of 30 nm.

On the other hand, studies aimed at promoting transistor architectures for reliable applications have taken advantage from the specificity of the antigen–antibody interaction to detect biomolecules in complex environments. The development of a new generation of biosensor transistors capable of detecting biosystems in biological fluids has led to relevant results. Immunosensing is undoubtedly a more efficient approach in terms of allowing low detection limits by biosensors, and EGOFETs have been shown to be effective for detection at very low concentration levels, as in the case of immunoglobulin-G, which experiences very effective binding capability with its related antibody, down to the zeptomolar (10^−21^) level [99]. EGOFETs have been demonstrated to be potentially effective for the detection of specific biomarkers in biological matrices, such as markers of pancreatic mucinous cysts in whole blood serum by using a single-molecule assay with a large transistor (SiMoT) device [100]; their effectiveness has been proven to be suitable for the detection of proteins, such as the C-reactive protein, in human saliva at a very low level using a specific self-assembling procedure to decorate the gate electrode by anti-CRP capturing antibodies [101]. Similarly, OECTs have shown promising performance in detecting biomolecules in complex fluids. For instance, Gentili et al. have shown that an OECT integrated with an immunoaffinity membrane can detect interleukin-6 within the picomolar range in the presence of interfering blood proteins [57].

The need for even more specific biosensing to overcome unspecific binding processes, involving undesired interfering biomolecules in complex matrices, has led to the development of more specific recognition systems, such as aptamers. Aptamers, which are synthetic nucleic acid sequences (i.e., short single-stranded DNA or RNA) capable of selectively binding with non-nucleic acid targets, allow to enhance biosensors’ selectivity due to the fact that aptameric binding is determined by its tertiary structure involving three dimensional structures. Relevant applications within aptameric sensing by biosensors with transistor structures are, for instance, an EGOGET for the detection of inflammatory cytokines in complex fluids [102] and an OECT device for the detection of thrombin in the presence of the most abundant blood protein (albumin) [103]. In the first case, tumor necrosis factor alpha (TNFα) has been detected at the picomolar range in a cell culture media containing 10% of serum in volume, using an easy functionalization of Au gate electrodes by the anti-TNFα aptamer solution. The second case deals with an OECT gated by a hierarchic structure made of polyethylene multi-layer graphene, Au nanoparticles and anti-Thrombin aptamers. The detection of thrombin at the picomolar level has been demonstrated by using a novel sensing parameter in the presence of albumin levels exceeding their physiological level in human blood.

### 3.4. Nucleic Acid Hybridization Sensors

Nucleic acid hybridization sensors can be useful for the identification of pathogens, genetic and viral diseases and tumors, and they can be involved for clinical purposes regarding diagnosis and monitoring and for pharmacological treatment and after surgery.

Bioelectronic organic transistors are a useful tool for nucleic acid sensing as they can provide the label-free single molecule detection of genomic biomarkers in peripheral fluids and allow for the detection of multiple biomarkers, paving the way to point-of-care diagnostics. Such devices are based on the hybridization of a single strand of nucleic acid with their complementary one, which belongs to the desired biomarker. Such a technique ensures specific recognition and great specificity.

Several solutions dealing with organic electronics have been proposed in the past few years in order to face the open challenges in the field of genomic biomarker detection. While most of them rely on EGOFET technology [100,104,105], other devices have been exploited for this purpose, such as OCMFETs [106] and OECTs [107].

**Figure 7:**
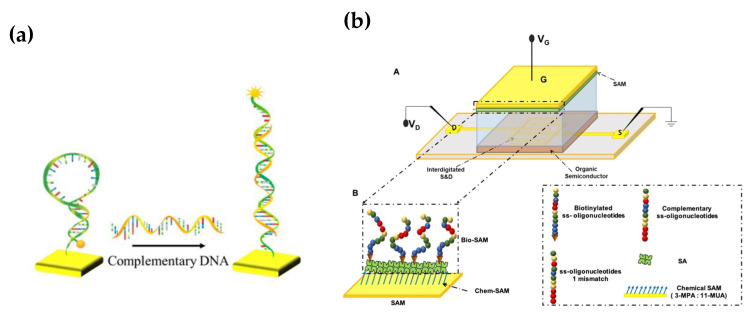
(**a**): Schematic of the hairpin-probe-sensing mechanism [106]. (**b**): (A) 3D schematic of the device. (B) Schematic of the gate functionalization with biotinylated single-strand oligonucleotides. Reprinted with permission from https://pubs.acs.org/doi/10.1021/acssensors.0c00694 [104]. Further permission related to the material excerpted should be directed to the ACS.

Proof of principle has been provided for the use of hairpin-shaped probes, in contrast to linear probes, on an OCMFET device for genomic biomarker recognition, following the working principle showed in Figure 7a. As a result, hairpin-shaped oligonucleotides have been successfully immobilized on a metallic surface, providing the device with sensitivity of 100 pM. The integration of hairpin-shaped probes with organic transistors paves the way to a new class of low-cost, portable and user-friendly devices with improved performance [106].

Other studies have focused on the detection of miRNA, of which takes part in many key processes and whose dysregulation might lead to several diseases. Both multiple sclerosis (miRNA-182) [104] and cancer (miRNA-21) [105] were considered to reach single-molecule LOD (10 zM) in the former case and 35 pM in the latter. Furthermore, Macchia et al. proved that a single molecule with a large transistor (SiMoT) platform, such as the one showed in Figure 7b already used for proteins, can be used for genomic biomarkers as well [104].

Single-molecule detection was also addressed for the detection of genomic markers for pancreatic mucinous cysts in whole blood serum through a label-free platform. Due to EGOFET technology, the detection of the protein markers MUC1 and KRAS was carried out correctly with an LOD, respectively, of 3 ± 2 molecules and 8 ±3 molecules in PBS and 6 ±2 molecules and 1 ±1 molecules in whole serum, which are good for clinical diagnostics. Macchia et al. provided a proof of principle for multi-analyte detection by the means of a SiMoT platform with several gates for proteins and genomic markers, paving the way to arrays for multiplexing [100].

At last, the functionalization process was investigated and the reduction in its complexity was addressed. A polydopamine (PDA) layer was taken into account, as the functionalities that are formed during the polymerization process from the neurotransmitter dopamine (DA) provide much easier functionalization, maintaining a great LOD ($100\times 10^{-15}\;M)$ [107].

### 3.5. Ions and pH-Detection Sensors

When it comes to ions and pH detection, organic electrochemical transistors are the devices of choice due to their inherent capability of translating ionic signals to electrical ones. In this perspective, several studies have been performed with the contribution of Italian research groups.

The research group from University of Bologna is particularly active on this topic. In 2018, Mariani et al. synthetized two pH-sensitive PEDOT composites by doping with pH dyes Bromothymol Blue (BTB) and Methyl Orange (MO) [58]. PEDOT:BTB in particular exhibited high sensitivity in the pH range from 1 to 9, and was therefore exploited to functionalize the gate electrode of an OECT, reaching a super-Nernstian response of 93 ± 8 mV/pH unit. In another work from the same research group, the authors exploited the integration of Ag/AgCl nanoparticles that act as a gate element directly in the PEDOT:PSS channel of an OECT, thus realizing a two-terminal OECT able to detect Cl^−^ in the range from 10^−4^ to 1 M [108]. Focusing on ion detection, Gualandi et al. then realized an OECT with an Ag/Ag_n_X-modified gate (X = Cl^−^, Br^−^, I^−^, and S^2−^) to be exploited as a potentiometric ion sensor [109]. Finally, Decataldo et al. exploited the transient-doping of PEDOT:PSS by molecular oxygen dissolved in solution to monitor the O_2_ level in cell culture media [65].

Concerning ion detection, a fruitful collaboration between researchers from the University of Brescia and the Max Planck Institute for Polymer Research (Mainz, Germany) allowed for the researchers to develop an organic electrochemical transistor complementary amplifier with ion detection capabilities of over five orders of magnitude with high resolution [110].

Moving away from OECTs, researchers from the University of Cagliari developed an OCMFET working as a reference-less pH sensor [111]. The authors exploited a plasma-activated Parylene-C layer as floating-gate functionalization, reaching *V_th_* variations of more than 1 V/pH unit. The reference-less architecture is foreseen to provide a viable platform for biocompatible, highly sensitive and low-cost applications.

### 3.6. Flexible and Wearable Sensors

In recent years, with the constant miniaturization of technology and sensors and the extreme decrease in their price, a strong interest has arisen from the textile and wearable industry. The idea to be able to constantly monitor every aspect of the human body with sensors inside clothes or to know the situation inside a car by having sensors in the fabrics of the car seats has collected the interest of companies now that it is always more feasible.

For this purpose, the flexibility of the substrate and of the device in general is almost always essential. The solution implemented can give the idea for the field of use: engineered fabrics will probably have a different use than flexible circuits on a flexible substrate.

Organic compounds have repeatedly shown their versatility regarding flexibility and biocompatibility, so they have been frequently implemented in devices for wearables.

Italy has a strong history in the textile and automotive industry: for this reason, the studies in this area have produced interesting results and it is a strong field in the literature production. A review from A. Nawaz et al. [86] has analyzed the specific impact of the geometry of the features on the flexible electronic devices with a comparison between planar and vertical OFETs and their advantages and disadvantages.

**Figure 8:**
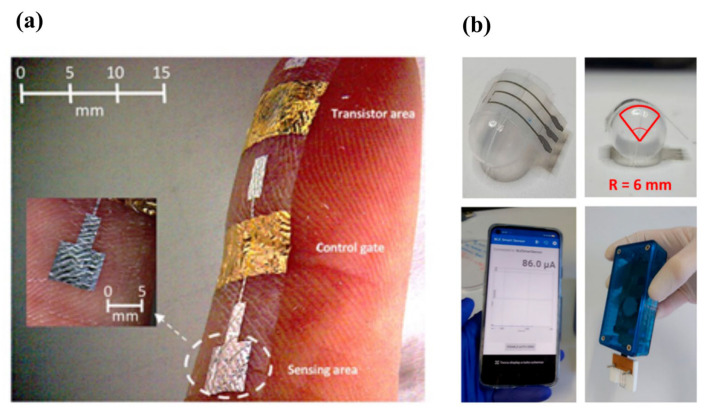
(**a**): OCMEFET placed on the skin of a finger, demonstrating its good conformability [113]. (**b**): OECT patterned on polyethylene naphthalate foil bent with a radius of 6 mm to mimic a real-life application; interfacing the sensor with a portable, handheld, battery-powered electronic readout from Elements srl company, wirelessly transmitting the current signal to a smartphone application [115].

A research group from the Department of Electrical and Electronic Engineering of University of Cagliari started by proposing an approach to evaluate what is the effect of mechanical deformation on the devices needed (for example, bending sensors opposed to strain-insensitive devices) and its related fabrication process [112], and fabricated with large area techniques a low-voltage Organic Charge-Modulated Field-Effect Transistor (OCMFET) [54]. They combined the OCMFET with a pyro/piezoelectric element for a low-cost and highly sensitive multimodal sensor [113] (Figure 8a) and integrated organic field-effect transistors into a Lycra^®^ glove for wrist-motion monitoring in real time [114]. Ultimately, they combined an OCMFET with a functional graphene electrode to be used as a chemosensor [68].

In the field of gas sensing, researchers from the University of Bologna fabricated an ammonia electrochemical sensor with poly(3,4-ethylenedioxythiophene) (PEDOT), iridium oxide particles and a hydrogel film: the device was tested as a wearable with sensitivity of 60 ± 8 µA/dec in a wide concentration range (17–7899 ppm) [116]. They exploited the combination of PEDOT and hydrogel film for the fabrication of an oxygen-sensing device on both glass and plastic substrates with (−12.2 ± 0.6) and (−15.4 ± 0.4) µA/dec, respectively, and a low power consumption [116] (Figure 8b).

Researchers at CNR Parma instead applied PEDOT:PSS on textile fibers and then an ion selective membrane for monitoring ions in sweat and were able to discriminate among the cations over the 10^−5^–1 M concentration range [117]. They also proposed a model for OECTs where internal configuration and geometrical parameters are not constant over time, and can better approximate the situation in bioelectronics in general, in particular in flexible and wearable devices [72].

Always from CNR Parma, a flexible and disposable OECT with PEDOT:PSS for the channels and a few layers of graphene for the gate electrode was exploited for the detection of Tamoxifen, with an LOD of 2.82 ng/mL for real-time measurements at zero-gate bias [1].

Biscarini’s research group worked on a flexible printed OECT for monitoring uric acid and demonstrated it in an artificial wound exudate with a limit of detection of 4.5 μM. They tested it both in phosphate-buffered saline and in complex solutions mimicking the wound exudate with reproducible results [70] (Figure 6).

For the bending sensing, Errico et al. fabricated a device with good electrical properties for low bending angles, making it promising as a complimentary bending sensor to the most diffused flex sensors [118].

To fabricate electronic textiles, Trovato et al. deposited vertically aligned carbon nanotubes (VACNT) and dispersed them in 4-dodecylbenzenesulfonic acid combined with silica-based sol-gel precursors, then compounded it with a polyurethane thickener and coated the cotton surfaces [119]. The composite showed a sheet resistance value less than 4.0 × 10^4^ ± 6.7 × 10^3^ Ω/sq and was tested as a humidity-sensing material.

In the field of modelling, Landi et al. proposed an accurate methodology that can quantitatively predict, for any organic semiconductor, the effects that the charge transport properties experience after an external deformation [120].

### 3.7. In Vivo and Cell Monitoring

One of the most treated topics nowadays in the literature research is the development of devices capable of carrying out biosensing monitoring in real-time, in vivo in an unobtrusive way, with minimal invasiveness and at a low cost. Organic electronics and transistor-based sensors can match many of these requirements, especially thanks to the properties of the materials they are made of, which are organic semiconductors. The organic electrochemical transistor (OECTs) is a special member of electrolyte-gated transistors (EGT): in its basic architecture, it consists of three electrodes (source, drain and gate) and the response is driven by controllable ionic and electronic currents. Source and drain contacts are interconnected by a semiconductive polymer—for example, the poly(3,4-ethylenedioxythiophene) polystyrene sulfonate (PEDOT:PSS)—whose dimensions define the features of the transistor channel. A gating bias modulates the drain current in the channel by injecting cations in the polymer backbone: any change in ion distribution inside the polymer results in a change in the electric current between the source and drain electrodes. OECTs are characterized by high transconductance which converts into a high amplifying capacity useful for applications in electrophysiology or health-monitoring in general [83].

**Figure 9:**
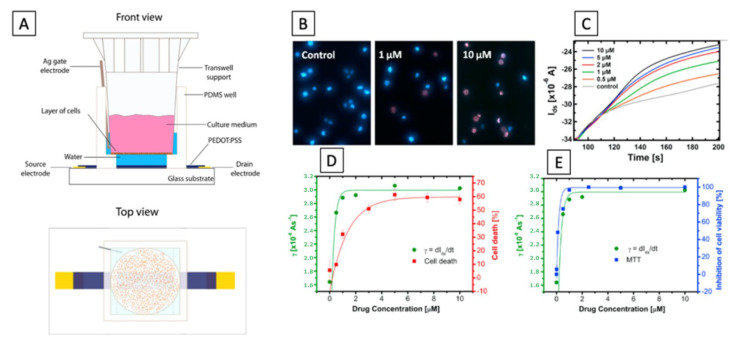
(**A**): Schematic of the front view and top view of a Tweel OECT. (**B**): Representative images of the fluorescence assay and the increasing doxorubicin concentration. The number of dead cells increase, increasing the drug concentration. (**C**): Kinetics of the source/drain current as a function of doxorubicin concentration. (**D**,**E**): Comparison between calculated γ parameter and results from the fluorescence and MTT assay [121].

We can find many efforts by Italian researchers in the biological/medical area for the development and optimization of organic devices allowing for the in vivo monitoring of cells, but also in the field of agriculture. The development of OECTs has allowed for a level-up in the monitoring and study of biological systems such as cells or molecules. In 2015, Romeo et al. [121] proposed an intriguing approach to study the effects of drugs on the phenomenon of cell death. They proposed an OECT coupled with a Tweel, on whose membrane cells (A549 cancer cells) were grown and exposed to the action of specific drugs. Information on cell death by apoptosis was extrapolated from the real-time monitoring of the current variation between source and drain. The treatment of cells with the drug (doxorubicin) induced a change in the structure and shape of the cells, which resulted in a change in the coverage of the Tweel membrane and consequently a change in the ion flux in the OECT channel. Figure 9 shows a schematic of the experimental setup used for the monitoring of cellular death dynamics, used in [121].

The same group studied cellular activity upon osmotic stress by using a T-Well-modified OECT sensor [122]. They demonstrate the possibility to detect cellular shrinkage in response to an imbalance of osmolyte in the cellular medium.

In 2022, the study of single cell adhesion by the means of impedance spectroscopy was reported [123]. Decataldo et al. reported the *in vitro* monitoring of virus kinetics for the real-time cytopathic effect evaluation [124]. Moreover, OECT devices are widely used for pH sensing, and in the medical field, this application is important to achieve a continuous monitoring of culture media pH [125] and wound infections [4]. In the first case, the continuous control of pH is needed to control the right and effective growth of cells in a culture media. In the second case—for example, in wounds—pH monitoring is needed for an early diagnosis of bacterial infections in wounds.

OECTs are promising electronic devices for biosensing and interfacing with biological systems, playing an important role for bio-signal monitoring [10], thanks to their low operating voltage (under 1 V) and high transconductance.

Monitoring the electrical activity of living organs is a well-known method to monitor their integrity and to prevent potential disease. Biological signals are characterized by low frequency (under 100 Hz) and a characteristic peak-amplitude voltage in the range from 10 μV to 10 mV.

Campana et al. [126] proposed a flexible and wearable OECT for electrocardiogram (ECG) recording *in vivo* and in *real-time*. OECTs were placed on the right arm and the Ag/AgCl gate electrode on the chest in proximity of the heart, resembling an Einthoven medical configuration. Exploiting OECT amplification and the skin/PEDOT:PSS impedance interface, cardiac ECG spikes were measured, which were comparable with signals acquired by commercial electrodes.

Organic-based devices applied as sensors for real-time, continuous and selective monitoring are important in healthcare, but also in the agriculture field as well. For example, the continuous monitoring of the levels of absorbed nutrients and water is important for the development of efficient and precise farming, which in turn, can play a fundamental role at the economic level.

The use of electrochemical devices, and in particular OECTs, has generated an improvement in the real-time monitoring of plant conditions thanks to the use of PEDOT:PSS, a well-known polymer for sensor applications characterized by an ionic and electronic current. The use of the OECT in the field of monitoring on plants has produced various scientific papers concerning the monitoring of water flows [127], ionic concentrations [5] or glucose inside plants [128], and recently, EGOFETs have also been exploited for monitoring bacterial infections in plants [129]. Gentile et al. in 2022 proposed an OECT integrated into the stem of a tomato plant for the real-time detection of the concentration and saturation levels of ions. This device consists of two functionalized textile electrodes inserted directly in the stem of the plant; the first is connected on both sides and constitutes the active part of the device (source, drain and PEDOT:PSS channel), and the second wire works as the gate electrode. Upon applying a positive voltage on the gate electrode, cation flux is generated from the gate to the PEDOT:PSS channel. This ion flux generates a variation in the current between source and drain; in this way, it determines ion concentration. The solution proposed in this article presents only one problem: it is not selective, but detects all the cations present in plant sap [5].

## 4. Neuromorphic Devices

### 4.1. Neuromorphic Devices Development and Modelling

In recent years, a major interest has been addressed by Italian groups working in organic electronics for the development and realization of devices based on polymers with neuromorphic functionalities. Neuromorphic devices are systems that can emulate the functions of synapses and neurons.

Two major approaches were followed: (i) the development of new devices from scratch, naturally endowed with neuromorphic functions [130,131] or (ii) the modification of already existing devices by the means of functionalization to trigger memorization abilities [2,132].

In both cases, significant attention has been paid in the improvement of performances and reliability [133], the selection (or modification) of different materials [2,134,135,136], geometries [131,137,138] and the identification of suitable fabrication techniques [3,139]. These design and development processes were obtained through a deep understanding of device physics and, in particular cases, polymeric chemistry, on which the working mechanisms are based on.

Beside these technological and materials aspects, Italian scientific production was addressed also in the development of new characterization protocols to investigate [140,141,142] and/or to trigger neuromorphic functions [10,130,143] in organic-based devices.

As already pointed out, Italian research on organic electronics can claim to have a valuable production on organic-based neuromorphic devices. In the following sections, a spectrum of working mechanisms, materials, fabrication methods and performances will be presented.

#### 4.1.1. Transistor-Based Neuromorphic Devices

Despite their large use in sensing applications, Electrolyte-Gated Organic Transistors (EGOT) have recently emerged as neuromorphic devices that exhibit synaptic plasticity and neuronal integration. The volatile nature of the electrical properties of this type of device limits in principle the possibility to memorize and retain long-lasting information. However, Italian groups, in line with colleagues from international institutions, have established functionalization protocols and/or device modifications to trigger neuromorphic functions.

As already pointed out in Section 3.1, EGOTs are usually classified as Electrolyte-Gated Organic Field-Effect Transistors (EGOFETs) and Organic Electrochemical Transistors (OECTs), depending on their operational regime.

In the first case, the selection of materials that constitute the device impedes ion permeation into the channel resulting in a final structure operating in field-effect mode. In OECTs on the contrary, the mechanism at the basis of the transistor operation is a doping/de-doping effect induced by ion permeation in the conductive channel by gate triggering. The capability of developing neuromorphic abilities is strongly related to the frequency with which trigger signals are delivered to the system and to the response time (τ) of the device—this is an efficient parameter for describing the rate of switching between the doped (on-state) and dedoped state (off-state).

A larger description of the transistor fabrication procedure, modelling and development is reported in Section 3.2.

#### 4.1.2. Redox-Based Memristive Devices

**Figure 10:**
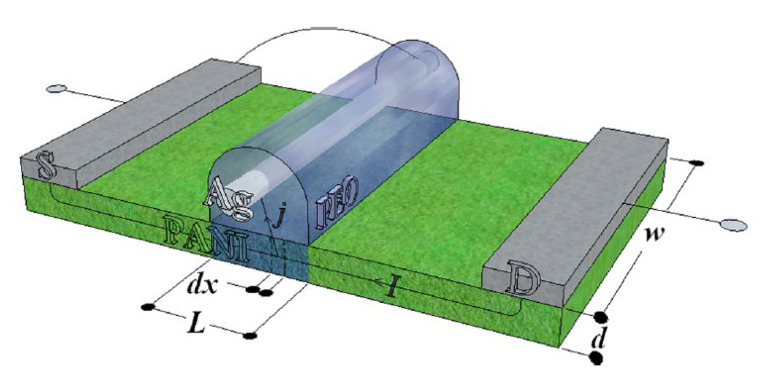
Scheme of the three-electrode electronic element based on polyaniline. Reprinted from Demin, V.A. et al. “Electrochemical model of the polyaniline based organic memristive device”. Journal of Applied Physics 116.6 (2014): 064507, with the permission of AIP Publishing [145].

Differently from the EGOTs case, redox-based memristive devices are a classical example of devices developed from scratch for the implementation of memorization ability and neuromorphic functionalities. In 2005, the realization of a three-electrode electronic element based on polyaniline–polyethylene oxide/LiCl with rectifying and memorizing behavior [144] was reported. This element, named Organic Memristive Device (OMD), constituted of a thin film of polyaniline (PANI) contacted by two metallic electrodes (source and drain) and placed in contact with a solid electrolyte in which a silver wire was inserted for serving as a gate (or reference) electrode (Figure 10).

*Materials and deposition techniques.* The predominant material in redox-based devices is polyaniline (PANI). The interest behind this conductive polymer arises from the possibility of combining multiple doping processes that ensure a large chemical, electrochemical and electronic spectrum of possibilities.

In Ref. [144], a PANI thin film, obtained by the Langmuir Schaeffer method, was placed in contact with a polyelectrolyte realized with polyethylene oxide (PEO) doped with LiCl (0.1 M). Despite the large interest in these devices, the preparation methodologies remained basically unaltered from 2005. Regarding the PANI layer, the most used deposition technique is the Langmuir Schaeffer method in which a monolayer, called the Langmuir film, is fabricated at the gas–liquid interface, and then it is horizontally deposited into a substrate. This latter is constituted of two evaporated chromium or gold electrodes on a glass insulant support. This deposition method allows for the realization of macroscale and microscale devices with a thickness that depends on the number of monolayers transferred on the substrate.

Typically, the subphase used is water; a solution containing PANI is deposited on the water surface with a microsyringe and it is compressed by two software-controlled barriers that control the surface pressure of the film. After solvent evaporation, the formed film can be transferred on the substrate by simply touching the interface.

Recently, a chitosan:polyaniline ink has been used in combination with aerosol jet printing for the realization of a PANI-based memristive device showing well-defined counterclockwise hysteresis/rectification and an enhanced durability [3].

In parallel, the composition of the solid electrolyte that is cast on the PANI layer has been widely investigated over the years to assess the role of the solute ions. Several authors reported the effects of variation of the concentration [146] and the dissolved salt [147] on the device’s performance. The clarification of these aspects allowed for a better understanding of the principle of operation of PANI-based memristive devices, from the perspective of improving their performance, stability and reliability.

The composition of the insulant matrix could be also adapted depending on the application’s needs. In [135], the authors reported the realization of a memristive device with a biocompatible solid polyelectrolyte based on pectin and chitosan, showing that the function of the device is preserved depending on pH conductions.

*Working mechanism and electrical characterization.* Regardless of the composition of the polyelectrolyte, the current–voltage behavior of these three terminal-PANI-based devices are attributed to the electrochemical reactions of the PANI under the solid electrolyte (4)

(4) $$PANI^{+}+e^{-}↔PANI^{0}$$

and this was afterwards demonstrated through UV-vis, FTIR and Raman spectroscopies and X-ray fluorescence analysis, of which clarified the role of the ions included in the polyelectrolytes used for the fabrication of the device [148,149].

**Figure 11:**
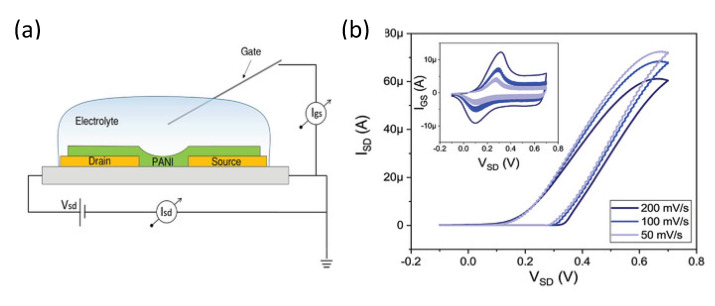
(**a**): OMDs scheme and electrical configuration: gold source and drain electrodes are in contact with a polymeric thin film of polyaniline (PANI). The latter is in direct contact with an electrolytic solution in which a silver wire is inserted as a gate electrode. Source and drain electrodes are biased with a V_SD_ voltage value, which induces resistivity variations in I_SD_ and I_GS_ currents; (**b**): I_SD_ response to V_SD_ variations as a function of the scan speed. In the inset, there are related I_GS_ responses. Copyright 2021 Wiley. Used with permission from Battistoni S. et al. “The role of the internal capacitance in organic memristive device for neuromorphic and sensing applications.” Advanced Electronic Materials 7.11 (2021): 2100494 John Wiley and Sons [130].

The typical electrical response of this kind of device (Figure 11a) is reported in Figure 11b, which has been obtained by biasing the PANI-based device with a triangular voltage sweep.

The marked hysteresis in the positive range of the voltage and the rectifying behavior in the negative one (Figure 11b) are the reflection of the electrochemical reactions occurring in the PANI channel (inset of Figure 11b) [150]. Due to this intimate connection between redox activity and conductivity variations, several predictive models have been developed for the simulation of the device operation. Initially in 2014 and subsequently in 2015, a detailed consideration of possible electrochemical processes occurring in the device resulted in models proposed by Demin at al. with qualitative and quantitative agreement with experimental data [145,151]. This latter model is based on the assumption that PANI oxidation or reduction reactions occur, respectively, at the potentials corresponding to the differences in activation energies (or energy barriers) for oxidation and reduction processes (roughly higher than 0.3 V and lower than 0.1 V, respectively) and that they can be expressed as Butler and Volmer equations.

**Figure 12:**
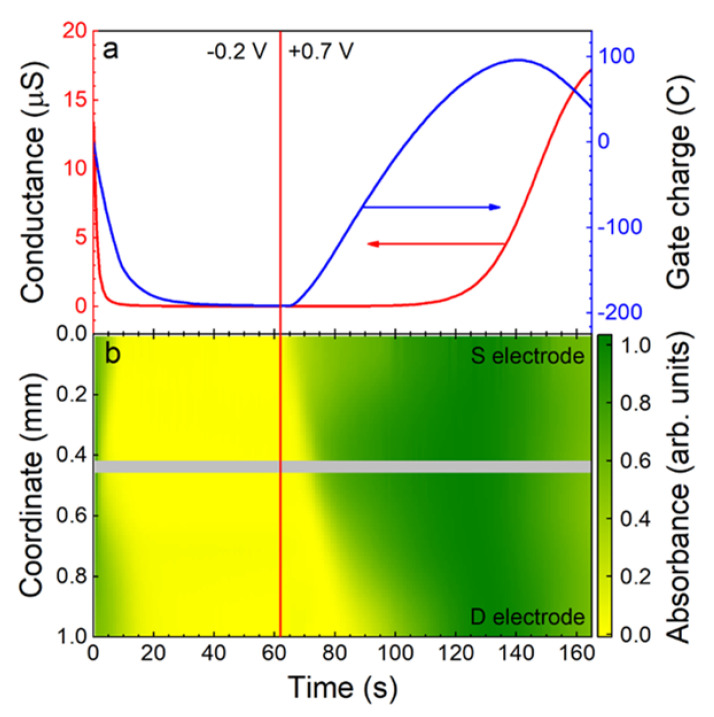
(**a**): Kinetics of changes in conductivity and gate charge in time under the applied voltages of −0.2 and +0.7 V. The red vertical line indicates the voltage change. (**b**): Heatmap of absorbance evolution in time within the active zone under applied voltages of −0.2 and +0.7 V. The grey horizontal line is the silver wire. The “source” electrode is on the top, the “drain” on the bottom of the panel [141]. Reproduced from D.A. Lapkin, A.N. Korovin, S.N. Malakhov, A.V. Emelyanov, V.A. Demin, and V.V. Erokhin, “Optical monitoring of the resistive states of a polyaniline-based memristive device”, Adv. Electron. Mater, 6, 2000511 (2020), © 2020 Wiley-VCH GmbH.

The working mechanism of these devices was inevitably associated with the evident color change of PANI due to its electrochromic propriety. The observation of these two independent variables (the color and the redox state obtained by electrical measurements) remained separated until Battistoni et al. [140] and Lapkin et al. [141] developed a spectroscopic and an optical method for correlating the actual conductivity of the PANI thin film with its color (Figure 12). The application of a constant voltage bias to OMDs induces a variation in the conductance which depends on the polarity, amplitude and duration of the pulse. The initial application of −0.2 V enhances the resistivity of the device (Figure 12a), promoting the formation of the yellowish leucoemeraldine (Figure 12b). The abrupt application of a positive bias induces in the system a marked increment in the conductivity and the transition of yellowish leucoemeraldine in the greener PANI-ES form.

The results of these two independent experiments can be considered as the demonstration of novel methods for the determination of the conductivity states, without the unavoidable electrical perturbations associated with the electrical measurement. In particular, the method reported in [140] shows a challenging perspective of acquiring redox-state information on a large number of PANI-based devices.

**Figure 13:**
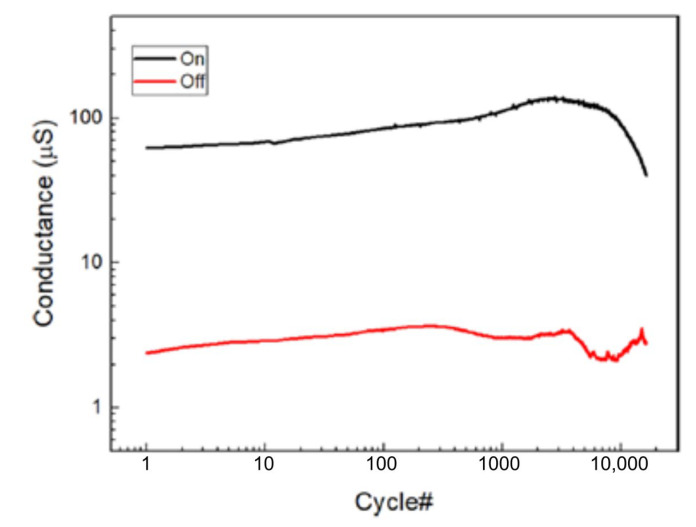
Endurance of microscale OMDs for different switching cycles from high-conductive (“on”) to low-conductive (“off”) states. Reprinted from Lapkin, D. A. et al. “Polyaniline-based memristive microdevice with high switching rate and endurance.” Applied Physics Letters 112.4 (2018): 043302, with the permission of AIP Publishing [133].

As already pointed out, OMDs could be realized both at micro and macro scales, taking advantage of Langmuir Schaeffer or Aereosol jet print methods. However, the reduction in the device’s dimension [133], the replacing of solid electrolyte with a liquid electrolyte [150] and the doping of PANI with polysaccharide [3] have been reported to be valuable methods for increasing the endurance and performance stability. In Figure 13, the switching stability of microscale OMDs was tested by applying a sequence of positive and negative voltage pulses interspersed with conductivity reading phases. The results of these acquisition steps are reported in Figure 13 as a function of the number of tested cycles. As clearly visible, the selected stimulation protocol is able to provide, in the OMDs, two resistive states (“on” and “off”) that correspond to the low and high conductive states. The devices switches from one state to the other up to 10,000 times prior to the face degradation processes [133].

#### 4.1.3. Filament-Based Memristive Devices

Besides from organic-based devices with a three-electrode configuration, a relatively newer class of polymer-based memristors is the two-electrode metal–insulator–metal structure.

**Figure 14:**
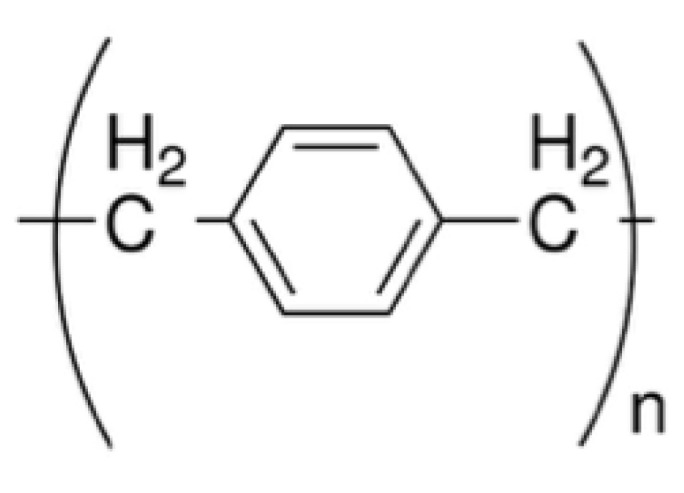
Parylene repetitive unit.

*Materials and deposition techniques.* The material of election for the realization of organic-based memristive elements is parylene (Figure 14) (poly-para-xylylene, or PPX), which can combine a simple and cheap production with its transparency and compatibility with flexible substrates. Regardless of its exploitation as a substrate or barrier layer [152], its use in the field of organic electronics is taking hold across multiple applications, including memristive devices [153]. In this scenario, a valuable example of a parylene-based memristor was reported by Minnekhanov et al. [154], in which a metal/parylene/indium tin oxide (M/PPX/ITO) structure was realized and electrically characterized. The parylene layers (~100 nm) were deposited on ITO-coated glass substrates (bottom electrode, or BE) by the gas phase surface polymerization method. Regardless of the electrical investigation, the authors deepened the role of the top metal electrodes (TEs), assessing the effect of different metals (Ag, Al or Cu) on the final device’s performance.

**Figure 15:**
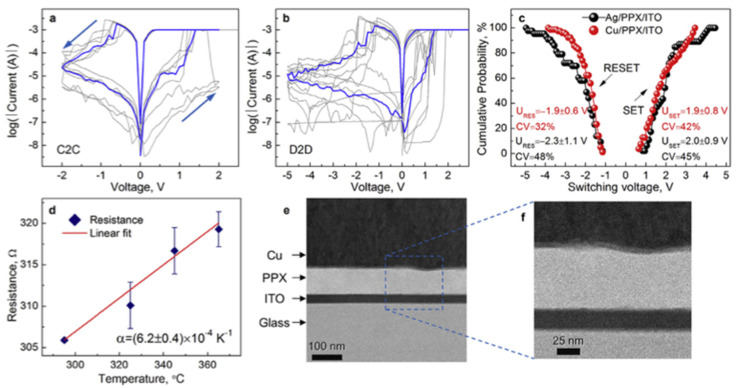
Electrophysical and structural characterization of the M/PPX/ITO structures. (**a**): I–V cyclic characteristics showing the typical bipolar resistance-switching behavior of the Cu/PPX/ITO sample during 7 cycles (cycle-to-cycle variability); the average curve is highlighted in bold. (**b**): I–V characteristics collected in 8 different Cu/PPX/ITO devices (device-to-device variability, the fifth of ten cycles is shown for each); the average characteristics are highlighted in bold. (**c**): Cumulative probabilities of U_SET_ and U_RESET_ switching voltages and their coefficients of variation (CV) for ~100 I–V cyclic characteristics measured in the samples with copper (red) and silver (black) top electrodes. (**d**): Temperature dependence of the low-resistance state of the Cu/PPX/ITO structure. (**e**): Cross-sectional TEM image of the Cu/PPX/ITO sandwich structure. (**f**): Enlarged image of the area highlighted by the rectangle in (**e**), showing roughness of the Cu/PPX interface [154]. Reprinted from Organic Electronics, Vol. 74, A.A. Minnekhanov, B.S. Shvetsov, M.M. Martyshov, K.E. Nikiruy, E.V. Kukueva, M.Y. Presnyakov, P.A. Forsh, V.V. Rylkov, V.V. Erokhin, V.A. Demin, and A.V. Emelyanov, “On the resistive switching mechanism of parylene-based memristive devices”, pages 89–95, Copyright (2019), with permission from Elsevier.

*Electrical characterizations and performances.* M/PPX/ITO memristive devices have generally good performance, with whatever metal used for the TE (Figure 15). However, the device structure that is considered mostly promising is the Cu/PPX/ITO structure, of which shows a good resistive range (R_off_/R_on_ value of ~10^3^), with endurance higher than 10^3^ cycles and the possibility of accessing 16 stable resistive states. Furthermore, such a structure has discrete cycle-to-cycle (C2C) and device-to-device (D2D) stability (Figure 15a and Figure 15b, respectively), further confirmed by the distribution of resistance-switching voltages U_SET_ and U_RESET_ (Figure 15c).

**Figure 16:**
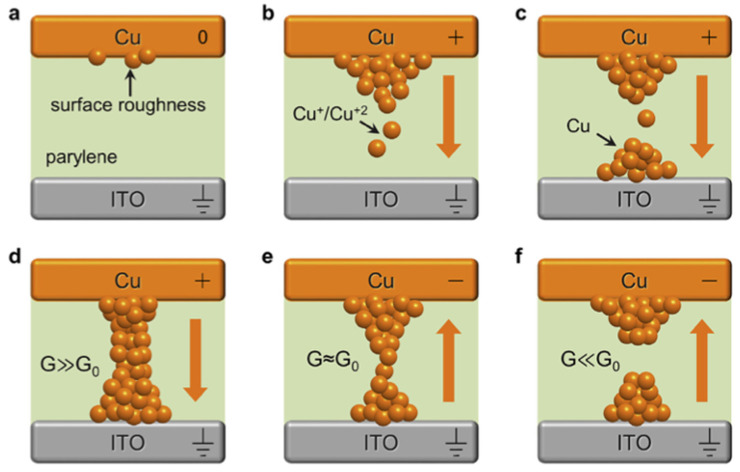
Schematic representation of the evolution of metal bridges (conducting filaments) in Cu/PPX/ITO memristive devices and the consequent quantum conductance effect. (**a**): Fragment of the pristine sandwich structure, having some surface irregularities on the top electrode. The orange pellets represent Cu atoms. (**b**): A positive voltage is applied to the top electrode of the structure; copper ions begin to move to the cathode (ITO) under the action of an electric field. (**c**): Copper ions reach the bottom electrode and reduce, so a conductive filament begins to grow. (**d**): The conductive filament is completely formed; quantized conductance is not observed. (**e**): A negative voltage is applied to the top electrode; copper ions begin to move backward to it. A quasi-point contact is formed, so the conductance is quantized, becoming approximately equal to G_0_. (**f**): The conductive filament has ruptured; conductance is much less than G_0_ [154]. Reprinted from Organic Electronics, Vol. 74, A.A. Minnekhanov, B.S. Shvetsov, M.M. Martyshov, K.E. Nikiruy, E.V. Kukueva, M.Y. Presnyakov, P.A. Forsh, V.V. Rylkov,, V.V. Erokhin, V.A. Demin, and A.V. Emelyanov, “On the resistive switching mechanism of parylene-based memristive devices”, pages 89–95, Copyright (2019), with permission from Elsevier.

*Working mechanism.* Minnekhanov et al. [154] suggested that the resistance-switching of such a parylene-based device is due to the formation of a conducting metallic filament in the dielectric film between the electrodes. To support this assumption, TEM investigations showing the formation of metallic bridges between the top and bottom electrodes of the M/PPX/ITO structures were reported (Figure 15e–f). The process of resistance-switching in the M/PPX/ITO structures is depicted in Figure 16. During the application of a positive voltage, the metal ions of the top electrode move into the polymer layer, migrate towards the bottom electrode and form the conducting filament (see Figure 16a–d). This filament remains thermodynamically stable until the biasing of the device with a negative voltage (Figure 16e–f), which induces the breaking of the thinnest part of the filament ruptures by Joule heating effect.

Understanding the operation mechanism is crucial to tailor the specific application of the memristive devices. In this view, the clarification of the resistive switching mechanism of M/PPX/ITO devices via the formation/dissolution of conductive filaments made these systems promising for the development of synapse-like networks.

### 4.2. Bio-Emulating Single Devices

The implementation of neuromorphic functions with organic devices has attracted a large interest in several Italian groups. The imitation of the brain’s ability to adapt, memorize and work in parallel has been a major task for researchers working in neuromorphic computing. These brain functions, also known as plasticity, are essentially localized in synapses that have the ability to strengthen or weaken over time in response to increases or decreases in their activity. These functions are commonly classified as short-term plasticity (STP) if the effects on the synapse last from a timescale of tens of milliseconds to a few minutes, and long-term plasticity if the duration is longer. Both the plasticity forms are crucial in the biological brain since they are considered important neurochemical foundations of learning and memory, and thus, their close emulation with electronic devices is considered a crucial step for the imitation of its efficiency and abilities.

#### 4.2.1. Devices for Emulation of Long-Term Plasticity

**Figure 17:**
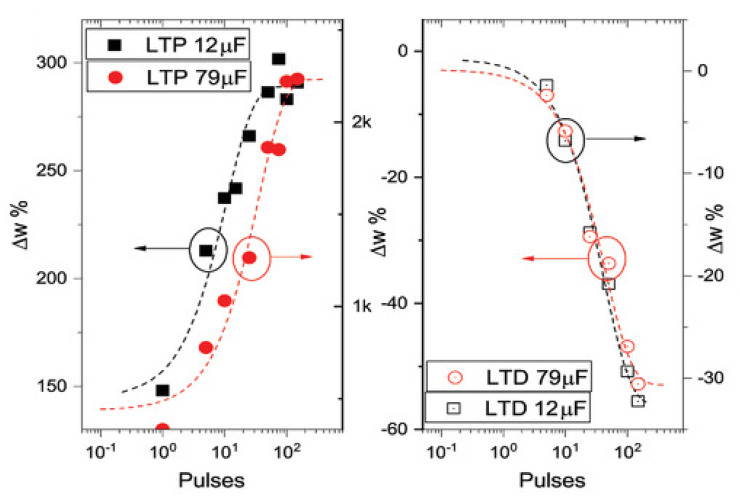
Emulation of synaptic long-term potentiation (LTP) and long-term depression (LTD) with OMD as a function of the number of delivered voltage spikes. Copyright 2021 Wiley. Used with permission from Battistoni, Silvia et al. “The role of the internal capacitance in organic memristive device for neuromorphic and sensing applications.” Advanced Electronic Materials 7.11 (2021): 2100494 John Wiley and Sons [130].

Being based on energy-barrier-activated processes that can be controlled by tuning the amplitude and duration of the (voltage) stimulus, OMDs have demonstrated to be naturally able to imitate the gradual increment or decrement of synaptic plasticity. In 2019, Battistoni et al. [155] reported that if stimulated with positive (or negative) voltage spikes, OMDs modified their internal conductivity depending on the number of incoming stimuli. The administration of many positive spikes induced a marked increment in the internal conductivity of the devices, while a negative stimulus reduced it. This effect, ascribable purely to the ability of OMDs to gradually increase their conductivity, was demonstrated in solid polyelectrolytes [155] (PEO+ LiClO_4_) and liquid electrolytes [130] (HCl 1M-Figure 17).

Furthermore, this increment or suppression in conductivity has been induced in OMDs also as a function of the frequency of the incoming stimuli and not of the spike’s polarity [155]. This feature is of particular relevance if considering that in natural synapses, a few seconds of tetanic stimulation (high-frequency sequence of individual pulses) enhances the synaptic strength while long periods of low-frequency stimulation induce the depression of the connection. When a single OMD experiences a high-frequency training routine, it increases its internal conductivity proportionally to the number and frequency of the incoming input signals. Instead, stimulation with lower frequency signals reduces the internal conductivity.

Despite the implementation of long-term plasticity with organic devices demonstrated by OMDs, several authors have reported interesting approaches for the implementation of this neuromorphic functionality by using PEDOT:PSS-based OECTs. In particular, the strategies explored a neurotransmitter-mediated operation [132] and modification in the composition of the PEDOT channel [156] and of the gate electrode [2]. In the first approach, an OECT made of a PEDOT:PSS channel is modulated by a gate electrode covered by the same polymer and in contact with dopamine exocytosed by PC-12 cells. The application of voltage pulses at the gate electrode induces dopamine oxidation, which results in a variation of the charge state of the gate electrode and a long-term modification of the conductance of the channel [132]. Interestingly, this system can develop a short-term modulation which is independent of dopamine concentration and long-term conditioning due to dopamine oxidation.

Another approach to trigger memorization ability in OECTs is based on the realization of PEDOT:PSS devices with a graphene-like gate electrode. This modification of the nature of the gate material allows to combine the OECT transduction properties with the ion-trapping capacity of carbon materials [2].

**Figure 18:**
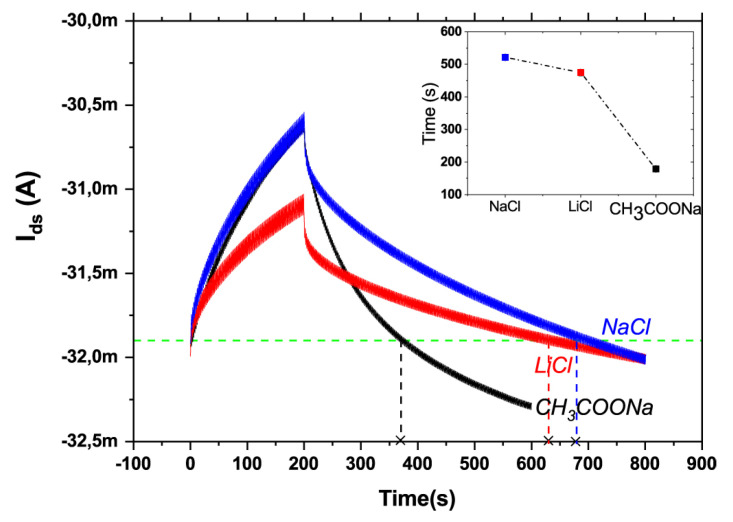
Response of OECT with a graphene-like gate electrode to a series of cumulative pulses (100 pulses at +1 V and >100 pulses at −0.5 V) as a function of the electrolyte composition. In the inset: plot of the time needed to reset the current to its initial value. Battistoni, Silvia et al. “Synaptic response in organic electrochemical transistor gated by a graphene electrode.” Flexible and Printed Electronics 4.4 (2019): 044002, DOI 10.1088/2058-8585 [2] © IOP Publishing. Reproduced with permission. All rights reserved.

The proposed working mechanism is based on the injection of cations in the PEDOT:PSS channel and a contemporary localization of anions in the proximity of gate electrode during the application of positive pulses [2]. Here, the morphological structure of the graphene-like material allows for ion penetration, intercalation and coordination by defects. Thus, during the successive application of negative pulses, cations and anions are gradually re-diffused back to the electrolytic solution for re-establishing the electro neutrality of the water solution. The efficiency of this effect is dependent on the anion’s size since it drives the probability of penetrating efficiently into the graphene material and the successive re-diffusion into the electrolytic medium. In fact, by varying the electrolytic composition and stimulating the OECT through the gate electrode with a series of 100 positive pulses (+1 V) followed by negative pulses (−0.5 V), the device shows response times that depend on the dimension and nature of the ions involved (Figure 18). Initially, by the application of positive pulses, the OECT shows a marked decrement in its internal conductivity due to the permeation of Na+ or Li+ ions in its PEDOT:PSS channel. Na^+^ intercalation seems to be more effective in de-doping the PEDOT with respect to the LiCl electrolyte. Successively, the application of negative pulses induces the re-diffusion of ions into the electrolyte with kinetics depending on the anion’s dimensions. Compounds containing Cl^−^ show slower kinetics with respect to the sodium acetate case [2].

#### 4.2.2. Devices for Emulation of Short-Term Plasticity

Despite the access to long-lasting memorization abilities and long-term plasticity features provides important advantages in the realization of artificial neural networks (ANNs), short-term neuromorphic functions are essential elements in biologic brain memorization processes and in the implementation of some unsupervised learning algorithms.

Short-term behavior of a whole organic artificial synapse has been demonstrated by Giordani et al. [9,131,137]. This system consists of two electrodes of PEDOT:PSS directly patterned through laser ablation onto PDMS (poly-di-methyl-siloxane) substrate and in electrical contact with an electrolytic solution containing dopamine (DA) and its interfering agents, ascorbic acid (AA) and uric acid (UA). The system shows a robust STP response which depends on the frequency of the incoming stimuli and the composition of the electrolytic solution [131,137].

In [157], authors reported displaying synaptic short-term plasticity that was obtained from the combination of supported lipid bilayers (SLBs) with OECTs for the development of biomimetic in vitro synapses. The presence of SLBs hinders ion permeation in the PEDOT:PSS channel, enlarging the response time of the systems regardless of the configuration of electrodes. Placing the gate electrode in the same plane (planar configuration) or in another plane (top orientation) seems instead to significantly impact the response time of OECTs, especially in the presence of the synthetic membrane. However, the STP operation was demonstrated also in electrolyte-gated organic synapstors (EGOSs) obtained by the use of a channel formed by Au nanoparticles (NPs) and pentacene.

The great merit of this approach is to show performances suitable to be interfaced to neurons with spike voltages compatible with action potential and with a dynamic response down to tens of milliseconds. The typical STP response of the EGOS shows a facilitation of (or increase in) the output or its depressing as a function of the incoming input’s frequency of the input spikes [158].

**Figure 19:**
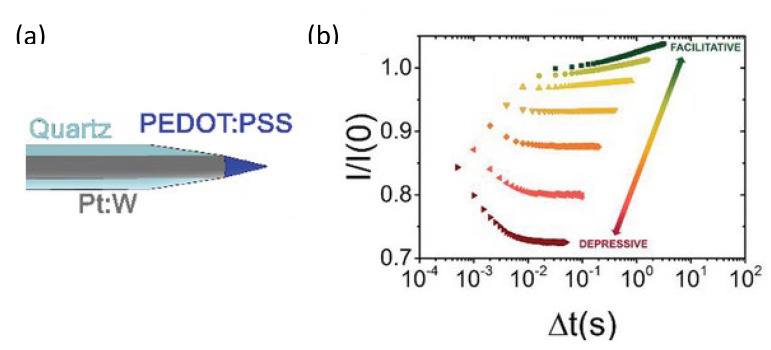
(**a**): Schematic representation of an implantable PEDOT:PSS artificial synapse; (**b**): expression of the facilitative or depressive STP behavior upon frequency variation. Reprinted ad adapted from [160]: Calandra Sebastianella, Gioacchino et al. “Implantable Organic Artificial Synapses Exhibiting Crossover between Depressive and Facilitative Plasticity Response.” Advanced Electronic Materials 7.12 (2021): 2100755 under a Creative Commons license.

Recently, reversible and tunable STP has been demonstrated in electrolyte-gated organic transistors (EGOTs), inverting the classical stimulation protocols. STP has been achieved in EGOTs by modulating the gate electrical field with a proper and selected pattern of stimuli while continuously monitoring the evolution of the current flowing in the polymeric channel. However, Di Lauro et al. [159] proposed a complementary protocol in which a steady potential is applied to the gate circuit and a series of voltage pulses are administrated between the source and drain electrodes. This approach ensures to obtain the STP depressive response governed by the combination of the two potentials delivered in the system, acting as two degrees of freedom regulating both the STP amplitude and the STP time constant at the same time [159]. In 2021, Calandra Sebastianella et al. [160] reported the realization of an implantable artificial synapse made of two PEDOT/PSS intracortical microelectrodes endowed with a STP response depending on the pre-synaptic frequency. In more detail, this structure is able to express facilitative or depressive STP behavior upon frequency variations (Figure 19).

#### 4.2.3. Devices for Spatio-Temporal Integration

Despite the large interest in the emulation of synaptic properties, the realization of integrated circuits mimicking different functions of biological neuronal networks is possible only by implementing neuronal features such as temporal or spatial integration. From a biologic point of view, these mechanisms are responsible for eliciting post-synaptic potentials in the presence of unsynchronized pre-synaptic activities or in the case of multiple, spatial, isolated synaptic action potentials, respectively.

Recently, Battistoni et al. [130] have reported the possibility of implementing both the temporal and spatial integration of incoming signals on a single OMD, thanks to the three-electrode configuration.

**Figure 20:**
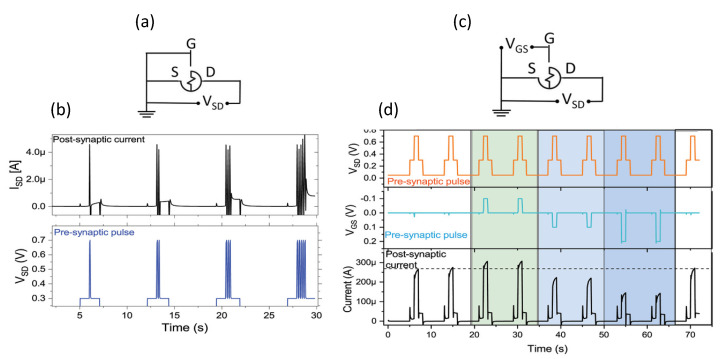
Spatio-temporal integration capabilities of OMDs: (**a**): Circuit-wiring diagram for operating the OMD for the temporal integration function. (**b**): Current output (black curve) and voltage profile (blue curve) used for the temporal integration. (**c**): Circuit-wiring diagram for operating the OMD for the spatial integration function. (**d**): Results of the spatial integration test. OMDs can integrate (bottom panel) two different “presynaptic” inputs (top and middle panels). Copyright 2021 Wiley. Used with permission from Battistoni, Silvia et al. “The role of the internal capacitance in organic memristive device for neuromorphic and sensing applications.” Advanced Electronic Materials 7.11 (2021): 2100494 John Wiley and Sons [130].

Authors demonstrated that a single OMD can integrate signals belonging to a single or multiple pre-synaptic pulses (Figure 20). The administration of a sequence of pulses from a single input (Figure 20a) results in eliciting the OMDs post-synaptic current whose value is proportional to the number of received spikes (Figure 20b). Analogously, in the presence of multiple incoming inputs (Figure 20c and orange and blue line in Figure 20d), OMD can spatially integrate synchronized pre-synaptic inputs with a final post-synaptic current that depends on the nature (excitatory or inhibitory–negative or positive pulses) or the intensity of the input. These trends mimic well the temporal and spatial integration of biological neurons.

### 4.3. Bio-Inspired Devices for Learning Algorithms

#### 4.3.1. Supervised Learning

In supervised learning paradigms, labeled datasets are used to train algorithms to classify and recognize data. These are obtained through weight adjustments until reaching complete convergence between the obtained input–output pair and the example input–output combinations of the network.

A typical example of supervised learning is an artificial neural network (ANN). ANNs are organizations of electronic elements in layers of non-linear threshold nodes that are connected to all nodes of previous and successive layers. This organization provides an adaptation of the propagation of the signal that depends on the “strength” (or synaptic strength) of connections between nodes that can be varied according to the applied training algorithm and on a defined threshold level. Despite the majority of artificial neural networks being realized at the software level, thanks to the easier implementation and training procedures, the software implementation of artificial neural networks has the significant limitation of being a power-hungry system due to the impossibility to perform parallel information. For this reason, several groups are working in the direction of the hardware implementation of artificial neural networks. A vast number of groups suggest the use of memristive devices as key elements in these neuromorphic applications, thanks to their synapse-mimicking properties, which candidate them as weight-function-varying connections [161,162]. Among the large class of ANNs, perceptrons are widely reported networks [163,164] that are originally designed for performing adaptation and classification tasks [165].

**Figure 21:**
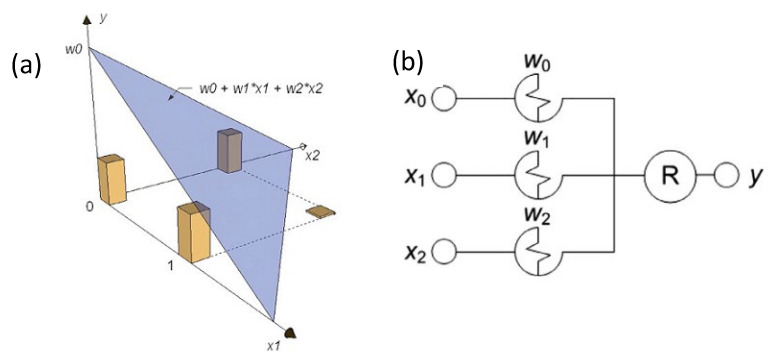
(**a**): Geometrical representation of the linear separability of objects, corresponding to NAND logic function [167]. Reprinted from Organic Electronics, Vol. 25, V.A. Demin, V. Erokhin, A.V. Emelyanov, S. Battistoni, G. Baldi, S. Iannotta, P.K. Kashkarov, and M.V. Kovalchuk, “Hardware elementary perceptron based on polyaniline memristive devices”, pages 16–20, Copyright (2015), with permission from Elsevier ; (**b**): Scheme of the elementary single-layer perceptron, based on organic memristive device [167]. Reprinted from Organic Electronics, Vol. 25, V.A. Demin, V. Erokhin, A.V. Emelyanov, S. Battistoni, G. Baldi, S. Iannotta, P.K. Kashkarov, and M.V. Kovalchuk, “Hardware elementary perceptron based on polyaniline memristive devices”, pages 16–20, Copyright (2015), with permission from Elsevier.

The first hardware realization of an ANN based on memristive devices was reported by Prezioso et al., using a metal-oxide device [166]. However, Demin and co-workers reported a few months later the realization of a single-layer elementary perceptron based on an organic memristive device [167]. In this case, a classification of linearly separable data according to NAND logic function was chosen as the task, as shown in Figure 21a [167].

The perceptron realized by Demin and co-workers consists of three inputs (x_i_, with i = 0,1,2); x_1_ and x_2_ correspond to variable features that the perceptron must classify and x_0_ is a fixed input, necessary for the proper classification (Figure 21b). These inputs are administered to three OMDs (W_0_, W_1_ and W_2_) acting as synaptic connections whose “weights” must be adjusted during the training procedure. A simplified version of the method of error correction [165] suggested by Rosenblatt was used for the training routine, for which the synaptic connections were adjusted according to the sign of the error between the actual and desirable values of the output signal.

The output value (y) is obtained by the sum of the weighted contributions of all devices (∑W_i_), plus a threshold value selected for discriminating one class from the other.

The selected function to be used during the classification was the NAND logic function; thus, to adapt the voltage/current behavior to the classical representation of logic functions, authors converted input and output signals into “1” and “0”. The graphical representation of the classification performed by the perceptron is shown in Figure 21a for the NAND function, in which three vectors of inputs (0, 0), (0, 1) and (1, 0) belong to class “1”, while the vector (1, 1) belongs to class “0”. The blue triangle in Figure 21b is the first-order plane that separates these objects, whose coordinates depend on conductivity values of OMDs. In [167], the author reported the importance of the selection of proper voltage values acting as input signals (0 and 1). Based on the experimental and simulation information [168], +0.4 V was chosen as logical “1” and +0.2 V as logical “0” to avoid any possible perturbations in the conductivity of OMDSs. On the contrary, for the weights updating, reinforcement and inhibition were performed with +0.7 V and −0.2 V pulses, respectively [168].

After successfully training the perceptron to perform the classification according to the NAND function, the authors successfully re-trained the same perceptron for the classification according to the NOR function.

**Figure 22:**
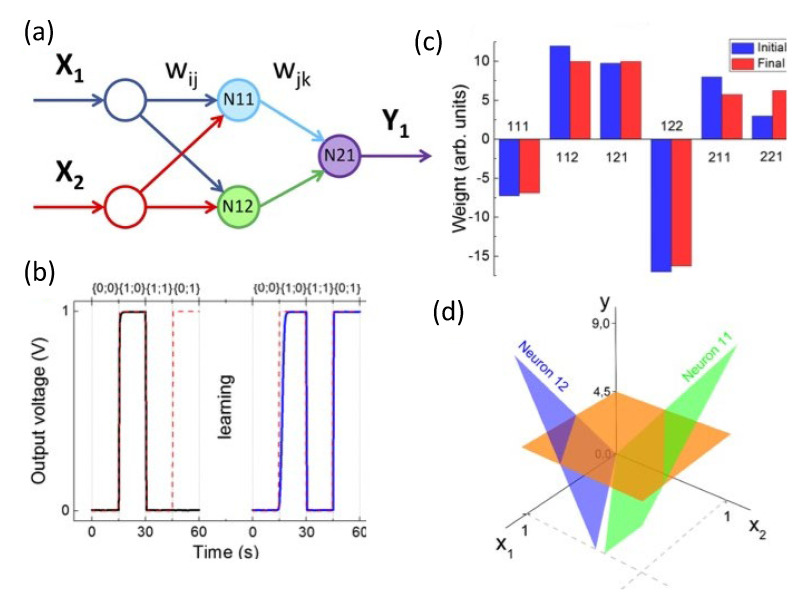
(**a**): Scheme of the double-layer perceptron; Experimental data. (**b**): Output signal within the epochs before (**left**) and after (**right**) training and expected output signal (dotted). (**c**): Synaptic weights and (**d**): corresponding feature plane partition (area above and below the plane y = 4,5 is the class “1” and “0”, correspondingly). Obtained separating planes are implemented by corresponding neurons in the first layer [169]. Reprinted from A.V. Emelyanov, D.A. Lapkin, V.A. Demin, V.V. Erokhin, S. Battistoni, G. Baldi, A. Dimonte, A.N. Korovin, S. Iannotta, P.K. Kashkarov, and M.V. Kovalchuk, “First step towards the realization of a double layer perceptron, based on organic memristive decices”, AIP Adv., 6, 111301 (2016) with the permission of AIP Publishing.

Despite the importance of the realization of a single-layer perceptron based on OMDs, to have access to higher levels of the classification (such as of linearly non-separable objects), it is necessary to increase the complexity of the ANN developing double-layer perceptrons. Emelyanov et al. [169] in 2016 reported the realization of a multi-layer perceptron with the capability of classifying data using the XOR logic function (Figure 22a).

The scheme contains two inputs (X_1_, X_2_), two neurons in the hidden layer (N11 and N12) and one output neuron (N22) [169,170]. The addition of layers in the scheme required, on the one hand, the realization of much more complicated circuits, and on the other, the development of supporting electronic circuits to minimally perturb the conductivity state of OMDs while acquiring the resistive states of devices. The authors proposed the simplified version of back propagation with the batch-correction learning algorithm [171] for the training procedures due to the cycle-to-cycle and device-to-device variability of OMDs.

The ANN realized by Emelyanov et al. was able to classify data belonging to the XOR logic function by adjusting the initial synaptic connections to match the desired final weights (Figure 22b,c). The presence of multi-neurons in the ANN gave rise to a more complex 3D representation of the classification process, as shown in Figure 22d, in which two output classes were divided by two planes.

Interestingly, Emelyanov et al. [169] proposed the double-layer perceptron as a valuable element for classifying objects not only in binary codes, but also analogue input signals.

These proposed examples of ANNs with different complexity and realized exploiting of the synaptic properties of PANI-based memristive devices suggest the possibility of developing larger networks with more abundant computing abilities, perspectively. This is partially due to the analogue and slow evolution of the internal conductivity of the organic memristive devices which avoid the use of more complex synaptic device organizations, such as crossbar arrays. Furthermore, this type of device develops multiple and stable internal resistive states that could be addressed during the network training. However, the variability of device-to-device performance represents a significant issue that could notably overcomplicate the prediction of the device behavior and, as a consequence, of the training routine of the networks, especially on higher scale ANNs and/or more demanding computation tasks.

#### 4.3.2. Unsupervised and Associative Learning

Despite the large interest in developing supervised learning algorithms and networks, a consistent number of groups dedicate their attention to the implementation of systems with unsupervised learning abilities. Unlike supervised learning, unsupervised learning employs unlabeled data to capture patterns and features encoded in the system. Among the possible paradigms for inducing unsupervised learning, the mechanism with a major highlight is spike-timing-dependent plasticity (STDP) [172]—a phenomenon by which the relative timing of spikes (pre- and post-synaptic spikes) affects the changes in synaptic strength (sign and magnitude) following the causal law.

Several papers reported the realization of practically ideal STDP-like learning with organic memristive devices [173].

**Figure 23:**
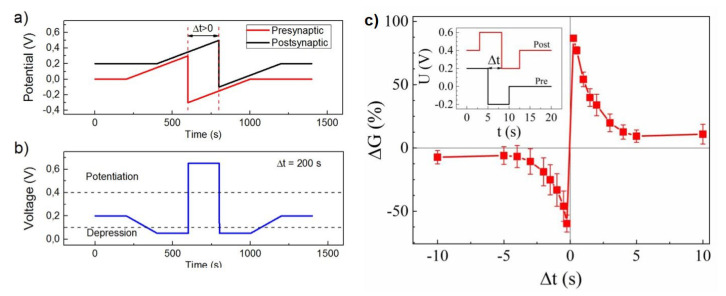
(**a**): Shapes of pre-synaptic (red line) and post-synaptic (black line) potential pulses. (**b**): Resulting voltage across the memristive element for the specific Δ*t* = 200 s value [174]. Reprinted from Microelectronic Engineering, Vol. 185, D.A. Lapkin, A.V. Emelyanov, V.A. Demin, T.S. Berzina, and V.V. Erokhin, “Spike-timing-dependent plasticity of polyaniline-based memristive element”, pages 43–47, Copyright (2018), with permission from Elsevier; (**c**): STDP window for the memristive-device-related conductance changes for different ∆t delay values. The inset shows the shapes of pre-synaptic (black) and post-synaptic (red) potential pulses [143]. Reproduced with permission from N.V. Prudnikov, D.A. Lapkin, A.V. Emelyanov, A.A. Minnekhanov, Y.N. Malakhova, S.N. Chvalun, V.A. Demin, and V.V. Erokhin, “Associative STDP-like learning of neuromorphic circuits based on polyaniline memristive microdevices”, J. Phys. D: Appl. Phys., 53, 414001 (2020). Copyright (2020) IOP Publishing, Ltd.

*STDP implementation in organic devices.* For STDP implementation, two electrical connecting schemes have been reported. Lapkin et al. reported the realization of the STDP algorithm with PANI-based OMDs by assigning at the gate electrode the role of the pre-synaptic input, and at the source electrode the role of the post-synaptic one [174] (Figure 23a). The form of the resulting pulses across the memristive element during the measurement for the delay time Δ*t* = 200 s is shown in Figure 23b. On the contrary, Prudnikov et al. [144] demonstrated the possibility of implementing the STDP setting source and drain electrodes as post- and pre-synaptic inputs (inset of Figure 23c). In both cases, post-synaptic pulses were applied with respect to the pre-synaptic pulses with a delay time Δ*t*, of which can have both positive and negative values.

Following both approaches, the authors demonstrated that OMDs are able to adjust their conductivity following the STDP window, expressed as the relative conductance change ∆*G*/*G* (∆*G*/*G* = |*G*_after_ − *G*_before_|/*G*_before_) vs. the time delay ∆t between pre- and post-synaptic pulses (Figure 23c).

The dependence, presented in Figure 23c, is in good agreement with the typical STDP window observed in biological systems [175].

STDP learning experiments have been conducted, even in the case of a device with a two-electrode configuration. Minnekhanov et al. reported in 2019 the development of STDP algorithms with parylene-based memristive devices [136]. In this case, the presence of two electrodes in the Cu/PPX/ITO memristive structure forced the assignation of the pre-synaptic input to the ITO layer and of the post-synaptic one to the top electrode (Cu).

**Figure 24:**
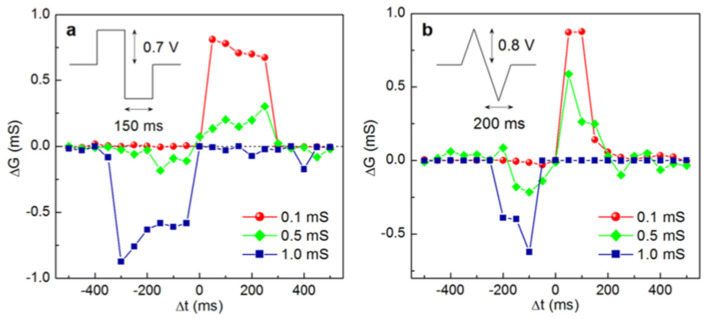
STDP window of Cu/PPX/ITO memristive structures (for various initial conductance values) obtained with heteropolar (**a**): bi-rectangular and (**b**): bi-triangular spike pulses shown in the figure insets. Post-synaptic spikes were applied after (before) pre-synaptic ones with a varying delay time Δ*t*. Every point of the curves is an average of 10 recorded experimental values [136]. Reproduced from A.A. Minnekhanov, A.V. Emelyanov, D.A. Lapkin, K.E. Nikiruy, B.S. Shvetsov, A.A. Nesmelov, V.V. Rylkov, V.A. Demin, and V.V. Erokhin, “Parylene based memristive devices with multilevel resistive switching for neuromorphic applications”, Sci. Rep., 9, 10800 (2019).

Variations in the memristor’s conductivity following the STDP rule could be obtained by stimulating the system with identical voltage pulses of bi-rectangular (inset in Figure 24a) or bi-triangular (inset in Figure 24b) shape. Varying the delay time Δ*t* between consecutives spikes, the Cu/PPX/ITO developed internal conductivity variations that obey to the biological STDP rule [173], depending on the initial conductance state.

*Pavlov’s dog experiment.* Associative learning is the process through which organisms acquire information and modify existing behaviors by establishing a correlation between contingent and contiguous stimuli as a consequence of repeated pairing of those events.

A valid example of associative learning is the classical conditioning experiment of Pavlov’s dog in which two stimuli (one unconditioned and a second neutral stimulus) are delivered to a system. The first one triggers a response of the system, while the second does not provoke any response. The contemporary administration of both stimuli induces a strong conditioning of the system that starts to respond even to the neutral stimulus that becomes a conditioned stimulus [176,177].

The implementation of this associative learning with organic devices has been demonstrated by multiple groups employing different devices.

**Figure 25:**
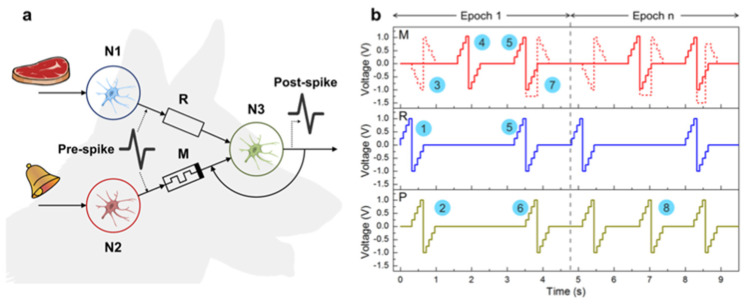
STDP-like learning memristive Pavlov’s dog implementation. (**a**): The electrical schematic diagram: N1—the first pre-neuron, spiking after the “food”-related stimulus; N2—the second pre-neuron, spiking after the “bell” stimulus; N3—the post-neuron, which spikes when the total input current exceeds the threshold; R—a resistor with a constant resistance value of R = 2 kΩ; M—a memristive element, initially in the R_off_ = 20 kΩ resistive state. A post-spike is generated unconditionally after a spike comes from N1 and under the condition that the memristor current exceeds I_th_ after a spike comes from N2. (**b**): An example of the spike pattern applied to the inputs of the scheme: 1—the initial pulse (first Epoch) on the resistor (R) (unconditioned stimulus), resulting in post-spike (P) 2, which in turn comes to the memristive device (M) as pulse 3 (dashed) in the inverted form; 4—the pulse on the memristive device, initially without post-neuron activity; 5—simultaneous pulses on the resistor and the memristive device, which result in post-spike 6 leading to the training pulse 7 (dashed); 8—a post-spike as a result of the conditioned stimulus when the training is completed (Epoch n, where n is equal to or above the number of epochs required for successful conditioning) [136]. Reproduced from A.A. Minnekhanov, A.V. Emelyanov, D.A. Lapkin, K.E. Nikiruy, B.S. Shvetsov, A.A. Nesmelov, V.V. Rylkov, V.A. Demin, and V.V. Erokhin, “Parylene based memristive devices with multilevel resistive switching for neuromorphic applications”, Sci. Rep., 9, 10800 (2019).

Minnekhanov et al. [136] reported the realization of a constructed network (simulating Pavlov’s dog behavior) consisting of two pre-synaptic neurons connected with a post-synaptic one (Figure 25a). In this work, the unconditioned stimulus (e.g., “food”) is delivered to the post-synaptic neuron through a resistor R and the neutral stimulus (e.g., a “bell”) is addressed to a memristive element (Cu/PPX/ITO).

The initial addressing of the unconditioned stimulus alters the post-synaptic response of the system. On the contrary, the administration of the neutral spike does not induce any response in the neuron. However, the contemporary application of neutral and unconditioned stimuli induces a resistivity change in the memristor (Figure 25b, spikes 5,6,7) that progressively leads to the conditioned response by which even the application of the “bell” signal alone results in the post-synaptic neuron spiking (spike 8).

An easier approach has been reported by Battistoni et al. in 2021 [130] where the authors reported the possibility of implementing the associative learning experiment of the Pavlov’s dog also in PANI-based OMDs. Taking advantage of the three-electrode configuration that allows for the contemporary stimulation of the devices with two input signals, the authors reported the possibility of conditioning the device response by the application of a neutral (“bell sound”) and unconditioned stimulus (“food”).

In summary, the two selected types of organic-based devices have demonstrated the capability of developing unsupervised and associative learning. Despite the two different geometries (i.e., two electrodes and three electrodes), PANI-based OMDs and PPX-based memristors have shown to adjust their internal conductive states, obeying to STDP learning rule and to the conditioning dynamics typical of the Pavlov’s dog experiment, if properly stimulated. The presence of three electrodes enriches the capability of the PANI device to integrate (synchronized or not) incoming signals and provide a higher number of degrees of freedom in the developing computation abilities in the system. On the contrary, the two-electrode devices have a more reproducible behavior and the developing of learning algorithms can be finely controlled by an accurate selection of acquisition circuits and of the incoming stimuli.

### 4.4. Devices for Coupling with Live Neurons

An essential step towards the realization of neuro-prostheses and brain-machine interface is to couple artificial electronic devices with neuronal cells [178]. Given the complexity of the topic, several Italian groups faced this situation by considering different aspects, among which include the influence of neurotransmitters, produced by neurons, on the characteristics of electronic systems and the evolving dynamics of the direct synaptic connection between electronics and living neurons.

In the first case [9], Giordani et al. reported the study of the influence of dopamine in the electrolyte solution on the characteristics of organic electrochemical transistors, based on PEDOT:PSS. Despite the lack of a direct interface with neuronal cells, in this work, the authors have demonstrated that the current response of the organic electrochemical device, made of two PEDOT:PSS electrodes, is selective for dopamine even without specific recognition moieties. The final system result is selective to dopamine against interfering agents produced from dopamine metabolic and catabolic pathways and it is particularly sensible to variations in dopamine concentration. This sensitivity has been successively supported by the work of Keene et al. where an organic electrochemical transistor, based on PEDOT:PSS, was exposed to the dopamine produced by nervous cells directly incorporated in a flow chamber [132].

This work is of particular relevance since it demonstrated that the proposed system response is dependent on the rates of dopamine release and diffusion. Furthermore, this biohybrid synapse follows the Hebbian model for synaptic connectivity.

Finally, Juzekaeva and co-workers reported the direct coupling of two live neuronal cells through organic memristive devices, using patch-clamp electrodes for connecting to neurons [179].

**Figure 26:**
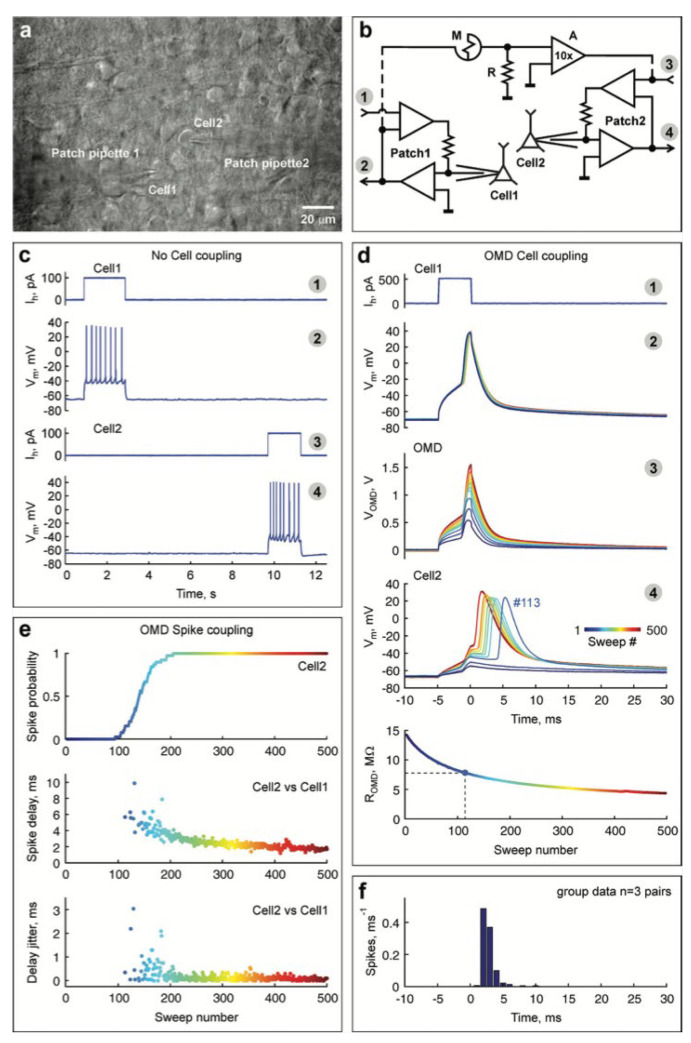
Activity-dependent coupling of neurons by the organic memristive device. (**a**): Infrared differential interference contrast microphotograph of a P7 rat brain slice with visually identified L5/6 neocortical cells (Cell1,2) recorded simultaneously. (**b**): Simplified electrical scheme of two patch-clamp amplifier headstages (Patch1,2): 1,3—patch-clamp holding inputs; 2,4—patch-clamp primary outputs; and an organic memristive device-based circuit (5 × 5 mm) connecting two neurons. (**c**,**d**): Traces of current-clamp recordings from Cells 1 and 2 before (**c**): and after (**d**): coupling through the organic memristive device. Traces 1–4 correspond to the inputs/outputs as labeled in (**b**). Note that prior to coupling through the organic memristive device (**c**), APs in either neuron failed to evoke responses in the other neuron, indicating that these cells were not connected by natural synapses. After the connection of Cells 1 and 2 through an organic memristive device (**d**), the efficacy of coupling progressively increases with each consecutive depolarizing step/AP in Cell 1. 500 traces (color coded by sweep #) are aligned with suprathreshold depolarizing steps delivered to Cell 1. Bottom plot, organic memristive device resistance as a function of the sweep #. Dashed lines indicate the first sweep when Cell 2 started firing. (**e**): Corresponding plots of the activity-dependent change in spike probability in Cell 2 (**top**), spike delay of Cell 2 from Cell 1 (**middle**) and spike delay jitter in Cell 2 (**bottom**). (**f**): Histogram of the spike delay in Cell 2 from Cell 1 calculated for three OMD-coupled cell pairs (777 spikes) [179]. Reprinted from E. Juzekaeva, A. Nasretdinov, S. Battistoni, T. Berzina, S. Iannotta, R. Khazipov, V. Erokhin, and M. Mukhtarov, “Coupling cortical neurons through electronic memristive synapse”, Adv. Mater. Technol., 4, 1800350 (2019), John Wiley and Sons, © 2018 WILEY-VCH Verlag GmbH & Co. KGaA, Weinheim.

To avoid any possible artifacts, non-connected pairs of cortical layer 5 pyramidal neurons were used for the patch-clamp recordings (Figure 26a). As evident, prior to the connection through any electronics device, action potentials (APs) evoked in one neuron failed to evoke any response in the other cell (Figure 26c), indicating that the latter were not connected by natural synapses. The successive coupling of these cells through an organic memristive device (Figure 26b), in a high resistive state (Figure 26d, bottom), was allowed to monitor the contemporary variation in the cell’s activities while acquiring the evolution of the internal resistance of the PANI-based OMD. Supra-threshold depolarizing steps (Figure 26d, plot 1) were administered to the “presynaptic” Cell 1 (Figure 26d, plot 2) that evoked only a subthreshold depolarizing response in the “postsynaptic” Cell 2 (Figure 26d, plot 4, blue color). This damping effect is due to the high initial OMD resistance (Figure 26d, bottom panel and Figure 26e, top panel). However, the successive depolarizing steps generate a marked lowering of the resistance of the OMD, which increases the voltage amplitude of the device responses (Figure 26d, plot 3) and the delivery of more intense stimuli to Cell 2 (Figure 26d, plot 4). When the OMD decreases its resistance by a factor of ≈2 (Figure 26d, bottom, sweep #113), the depolarizing response in Cell 2 reaches the AP threshold (≈−40 mV) and it starts reliably to fire (Figure 26d, plot 4, sweep #113 onwards and Figure 26e). It is worth noting that the firing probability of Cell 2 (Figure 26e, top), the spike coupling between neurons (Figure 26d, plot 4 and Figure 26e, middle plot) and the reduction in the jitter of AP delays (Figure 26d, plot 4 and Figure 26e, bottom plot) were undoubtedly associated with the activity-dependent evolution of the OMD internal resistance [180]. The analysis of the delay times between cells spiking (Figure 26f) suggests a good agreement between the characteristic timing of the AP commutation through the OMD synapse and the one expected in natural excitatory synapses.

**Figure 27:**
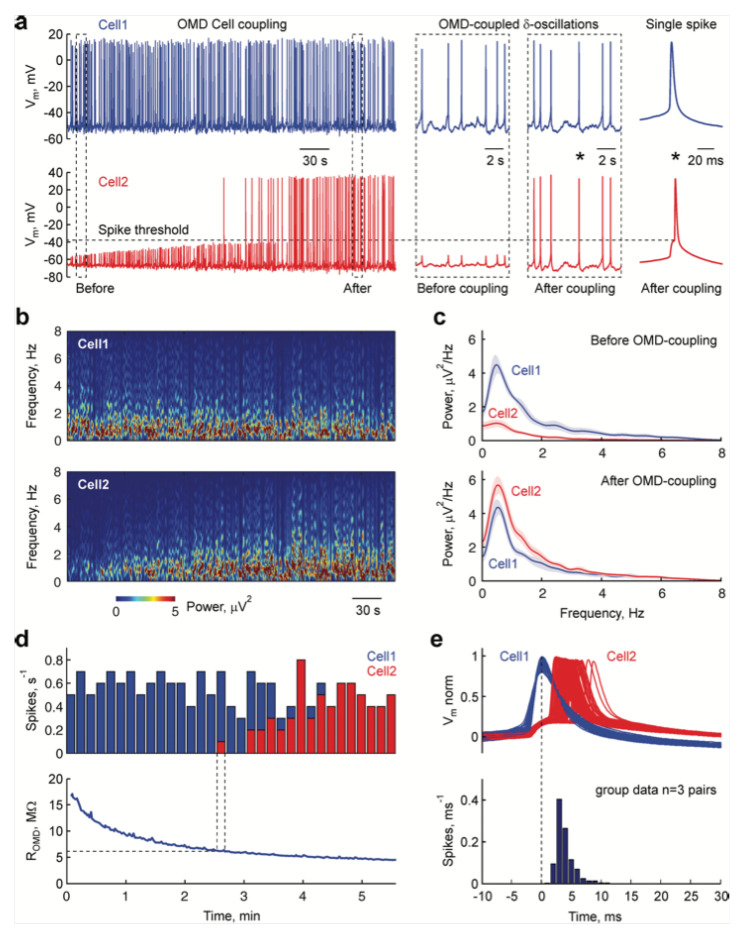
Synchronous oscillations in the natural neuron network where two cortex neurons were coupled through the organic memristive device. (**a**): Current-clamp recordings from Cell 1 (blue) and Cell 2 (red). Parts of traces outlined by dashed boxes before and after spike coupling through organic memristive device are shown on expanded time scales on the right. The horizontal dashed line indicates Cell 2 spike threshold. (**b**): Corresponding membrane potential spectrograms in Cells 1 and 2. (**c**): Power spectrum density plots of the membrane potential in Cells 1 and 2 before (**top**) and after (**bottom**) spike-coupling through the organic memristive device (confidence interval is shadowed; n = 3 pairs). (**d**): Frequency of spikes (top) in Cell 1 (blue) and Cell 2 (red) calculated for the 10 s bin intervals from the recordings shown in a and the corresponding values of the organic memristive device resistance (**bottom**). Dashed lines indicate the onset of spike-coupling between Cells 1 and 2. (**e**): Example of 65 normalized spikes (**top**) recorded in Cell 1 (blue traces) and Cell 2 (red traces) and the histogram of the spike delay in Cell 2 from Cell 1 (bottom), data from three organic memristive device-coupled cell pairs (633 spikes) are pooled together [179]. Reprinted from E. Juzekaeva, A. Nasretdinov, S. Battistoni, T. Berzina, S. Iannotta, R. Khazipov, V. Erokhin, and M. Mukhtarov, “Coupling cortical neurons through electronic memristive synapse”, Adv. Mater. Technol., 4, 1800350 (2019), John Wiley and Sons, © 2018 WILEY-VCH Verlag GmbH & Co. KGaA, Weinheim.

Subsequently, Cell 1 was continuously depolarized by the injection of the constant current to allow for spontaneous firing (Figure 27a, blue trace). This continuous activity of the neuron induced a gradual decrease in resistance of the OMD (Figure 27, bottom) that led to an activation of the Cell 2 response (Figure 27a, red trace) that started firing APs in synchrony with Cell 1 (Figure 27a,d). This neuronal synchronization during spontaneous activity occurred in the δ-frequency range (0.56 ± 0.04 Hz, n = 3 cell pairs; Figure 27b,c) that is characteristic of the slow-wave cortical activity during deep sleep [181]. The results reported by Juzekaeva and co-workers represent the first experimental demonstration of the unidirectional, activity-dependent coupling of living neurons through a device with synaptic functionalities. The key point of this research is the demonstration that the activity-dependent response of the organic memristive device well mimics and resembles the one of the natural excitatory synapses. This similarity is so noticeable that the integrated system between active cells and OMDs shows the possibility of controlling the level of coupling by neuronal activity patterns and efficiently supporting neuronal synchronization.

## 5. Conclusions

In conclusion, in this work we summarized the results achieved by the Italian research community concerning Organic Bioelectronics. The latest developments have been reviewed together with older milestone papers that have been trendsetting over the past two decades. From this analysis, it clearly emerges how Organic Bioelectronics is a growing research field with important contributions from Italian researchers. Italian research groups working on Organic Bioelectronics are in fact among the most prolific in terms of publications. As highlighted in this review, over the past few years, groups from different institutions contributed to the advancement of the field with the development of devices ranging from electrolyte-gated organic transistors to organic memristive devices, and with their successful integration with biological systems. The biocompatibility and performances granted by the use of organic materials have attracted growing attention, and this trend is expected to continue in the future, with novel materials and technologies continuously being developed. Current studies are more and more focused on addressing the different challenges still limiting the widespread employment of these technologies, such as device performances, stability and scalability to an industrial level. Innovative techniques such as 2D/3D printing are explored for the device fabrication together with well-established microfabrication techniques, paving the way for the future realization of large-scale mass production of low-cost and high-performance devices. The results obtained in terms of bio-chemical sensing and bio-interfacing in the mimicking of neuronal activities and in the hardware implementation of supervised and unsupervised learning algorithms may herlald the future realization of systems implementing more than one of these functions, opening possibilities for unforeseeable applications.

Of course, research activities in Italy are strongly correlated with similar approaches in other countries. In particular, organic field-effect transistors for sensor and neuromorphic applications go in parallel and sometimes with the involvement of partners from the UK, Germany, France and Netherlands, indicating that this line has the highest priority within EC scientific strategies.

Regarding neuromorphic systems, based on memristive devices, it is to note that significant advantages were achieved by groups of R. Waser (Germany), J.J. Yang (USA) and D.B. Strukov (USA). However, these groups work mainly in the field of inorganic devices (the topic is beyond this review). In the case of organic implementations of memristive-based neuromorphic systems, Italian groups have several priorities, such as systems, allowing supervised and unsupervised learning, bio-plausible plasticity, the hardware reproduction of neural systems parts and coupling with live neurons.

As a final remark, we can argue that the “Italian bioelectronics wave” is quite active and creative and covers different scientific fields from chemistry, material science, physics and engineering to develop biosensors and memristors for biomedical, agriculture and in vivo applications.

## Data Availability

Not applicable.
